# Cerebral amyloid angiopathy and amyloid load distribution detected on amyloid–positron emission tomography: A systematic review and meta-analysis

**DOI:** 10.1093/esj/23969873251349657

**Published:** 2026-01-01

**Authors:** Aikaterini Theodorou, Konstantinos Melanis, Athanasia Athanasaki, Lina Palaiodimou, Maria-Ioanna Stefanou, Panagiota-Eleni Tsalouchidou, Efthimios Vassilopoulos, Anastasios Kouzoupis, Marios Themistocleous, Georgios P Paraskevas, Elias Tzavellas

**Affiliations:** Second Department of Neurology, “Attikon” University Hospital, School of Medicine, National and Kapodistrian University of Athens, Athens, Greece; Second Department of Neurology, “Attikon” University Hospital, School of Medicine, National and Kapodistrian University of Athens, Athens, Greece; Second Department of Neurology, “Attikon” University Hospital, School of Medicine, National and Kapodistrian University of Athens, Athens, Greece; Second Department of Neurology, “Attikon” University Hospital, School of Medicine, National and Kapodistrian University of Athens, Athens, Greece; Second Department of Neurology, “Attikon” University Hospital, School of Medicine, National and Kapodistrian University of Athens, Athens, Greece; Department of Neurology & Stroke, Eberhard-Karls University of Tübingen, Tübingen, Germany; Hertie Institute for Clinical Brain Research, Eberhard-Karls University of Tübingen, Tübingen, Germany; Second Department of Neurology, “Attikon” University Hospital, School of Medicine, National and Kapodistrian University of Athens, Athens, Greece; First Department of Psychiatry, “Aiginition” Hospital, School of Medicine, National and Kapodistrian University of Athens, Athens, Greece; First Department of Psychiatry, “Aiginition” Hospital, School of Medicine, National and Kapodistrian University of Athens, Athens, Greece; Department of Neurosurgery, Agia Sofia Children’s Hospital, Athens, Greece; Second Department of Neurology, “Attikon” University Hospital, School of Medicine, National and Kapodistrian University of Athens, Athens, Greece; First Department of Psychiatry, “Aiginition” Hospital, School of Medicine, National and Kapodistrian University of Athens, Athens, Greece

**Keywords:** Cerebral amyloid angiopathy, positron emission tomography, amyloid load, Alzheimer’s disease, hypertension, intracerebral hemorrhage%

## Abstract

**Introduction:**

There are limited data regarding the amyloid positron emission tomography (PET) imaging among patients with Cerebral Amyloid Angiopathy (CAA). We sought to assess the amyloid load distribution detected on amyloid-PET among CAA patients compared to patients with Alzheimer’s Disease (AD), patients with hypertension (HTN) related hemorrhage (ICH) and healthy controls (HC).

**Patients and methods:**

A systematic review and meta-analysis of published studies with available data on global and regional amyloid-PET uptake was conducted. Comparisons with respect to amyloid load distribution were investigated using random-effects models based on the ratio of mean (RoM) amyloid-PET uptake. RoM < 1 and RoM > 1 indicate lower and higher global or regional amyloid-PET uptake in CAA compared to another population, respectively.

**Results:**

We identified 16 cohorts, comprising 271 CAA patients (mean age: 72 years; women: 46%) versus 130 AD patients (mean age: 73 years; women: 44%), 180 patients with HTN-related ICH (mean age: 66 years; women: 36%) and 61 HC (mean age: 71 years; women: 46%) with available data on amyloid-PET. Global amyloid PET ratio differentiated CAA from AD [RoM: 0.93; 95% CI: 0.90–0.96; *p* < 0.0001], HTN-related ICH [RoM: 1.25; 95% CI: 1.20–1.31; *p* < 0.0001], and HC [RoM: 1.26; 95% CI: 1.23–1.29; *p* < 0.0001]. Occipital amyloid-PET uptake [RoM: 1.20; 95% CI: 1.15–1.26; *p* < 0.0001] was higher in CAA compared to HTN-related ICH, and Occipital-to-global [RoM: 1.05; 95% CI: 1.03–1.07; *p* < 0.0001] ratio of amyloid-PET uptake differentiated also CAA from AD.

**Conclusions:**

CAA is characterized by a distinct amyloid-PET burden and distribution compared to AD patients, patients with HTN-related ICH and HC. These findings may contribute to the design and conduct of future randomized controlled clinical trials, aiming to treat CAA at preclinical stages.

## Graphical Abstract

**Graphical abstract:**
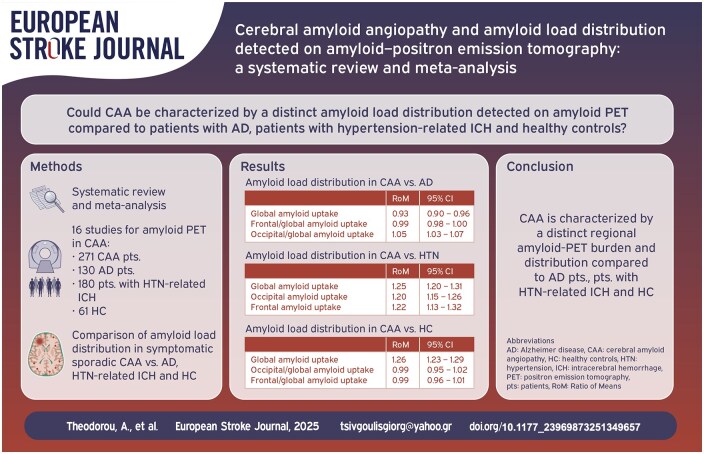

## Introduction

Sporadic Cerebral Amyloid Angiopathy (CAA) represents a distinct small vessel disease, characterized by the progressive microvascular deposition of amyloid-β (Aβ) in leptomeningeal and cortical vessels.^1^ Boston criteria have been developed to allow the possible or probable in vivo diagnosis of the disease, in the absence of characteristic neuropathological evidence from an invasive brain biopsy or post-mortem examination.^2,3^ Interestingly, recent studies propose that subclinical pathophysiological alterations begin and progressively accumulate almost 30 years before the first clinical manifestations of hemorrhagic lesions.^4^ Earlier detection of CAA pathology is crucial, since intervention with novel disease-modifying agents at preclinical stages could maximize the therapeutic benefit.

Alzheimer’s disease (AD) pathology frequently co-occurs with CAA, approximately in 80%–90% of CAA patients.^1,5–8^ Distinguishing possible contributions to the cognitive decline of the overlapping underlying pathologies remains a huge challenge. Cerebrospinal fluid (CSF) biomarkers demonstrate a distinct pattern among patients with CAA compared to those with AD or healthy controls.^9^ However, vascular amyloid deposition, characteristic for CAA cannot be disentangled from the parenchymal amyloid deposition of AD, even with advanced neuroimaging methods, including amyloid positron emission tomography (PET).^10^

PET imaging with^11^C-Pittsburgh compound B (^11^C-PiB) or ^18^F-florbetapir tracers may provide an opportunity for an earlier and more accurate diagnosis of the disease or a significant key to differentiate the underlying etiology of cognitive impairment.^11^ Preliminary studies have demonstrated a distinct pattern of amyloid load distribution, with the characteristic global cortical amyloid load and occipital-to-global regional distribution.^12–14^

In terms of these observations, we performed a systematic review and meta-analysis of the available literature data regarding the evidence of PET imaging on CAA patients. We sought to investigate and assess the global cortical amyloid load and different regional distributions as well among CAA patients compared to AD patients, patients with hypertension-related intracerebral hemorrhage (HTN-ICH) and healthy controls (HC).

## Subjects and methods

### Data availability statement

The datasets used and analyzed during this systematic review and meta-analysis are included in this manuscript and its Supplemental Material. More detailed datasets are available from the corresponding author on reasonable request.

### Standard protocol approvals, registrations, and patient consents

The pre-specified protocol of the systematic review and meta-analysis has been registered in the International Prospective Register of Ongoing Systematic Reviews PROSPERO (CRD42O25646130).

The meta-analysis is reported according to the updated Preferred Reporting Items for Systematic Reviews and Meta-Analyses (PRISMA) guidelines^15^ and was written according to the Meta Analysis of Observational Studies in Epidemiology (MOOSE) proposal.^16^ The systematic review and meta-analysis did not require an ethical board approval according to the study design.

### Data sources and database searches

A systematic literature search was conducted to identify eligible studies evaluating studies reporting evidence on brain PET among patients with CAA, and/or AD patients, and or patients with HTN-related ICH and/or HC.

The literature search was performed independently by three reviewers (A.T., K.M., G.T.). We searched PUBMED and Scopus, using search strings that included the following terms: “Cerebral Amyloid Angiopathy,” and “Positron emission tomography”; the complete search algorithm used in PUBMED and Scopus is described in the Supplemental Methods. The details on database search are included in the Methods section of the Supplemental Material. No language or other restrictions were applied. Our systematic literature search was conducted up to January 25,2025 for each electronic database. Additional manual search included conference abstracts and bibliographies of candidate studies and recent systematic reviews for a comprehensive literature search.

### Study selection

We included full-text, published studies, involving: (1) patient cohorts diagnosed with CAA based on original, modified (v1.5) and v2.0 Boston criteria and AD based on NIAAA or NINCDS-ADRDA ^2,3,17,18^; (2) available data on the amyloid load distribution, as detected on the amyloid-PET among patients with CAA and/or AD patients and/or patients with deep ICH related to HTN and/or HC; (3) adult patients (⩾18 years old).

We excluded studies that did not report data with regard to amyloid load distribution, as detected on the amyloid-PET among patients with CAA, studies with overlapping data, and studies reporting on ⩽5 patients. Editorials, commentaries, and narrative reviews were also excluded.

### Data extraction

Three authors (A.T., K.M., L.P.) independently reviewed the retrieved articles as summarized in the Supplemental Methods section of Supplemental Material and any disagreements were resolved after discussion with the senior (E.T.) and corresponding (G.T.) authors.

The following information were extracted: name of the study, first author and year of the publication, study design, and data collection interval, mean age, sex distribution, total number of study participants, tracer used (^11^C-PiB or ^18^F-florbetapir) for the PET imaging and the method of quantifying tracer uptake [Distribution Volume Ratio (DVR) or Standardized uptake value ratio (SUVR)]. These two tracers are expected to provide comparable results in the evaluation of amyloid burden, taken into account previous study showing linear association between mean global cortical ^18^F-florbetapir retention and ^11^C-PiB uptake values in CAA cohort.^19^

### Primary analyses

An aggregate data meta-analysis was performed with the inclusion of all the eligible cohort studies. We primarily assessed the ratio of means (RoM) as the effect measure, which was calculated as the ratio of mean amyloid global or regional uptake between two groups of interest. We sought to meta-analyze data on amyloid global or regional load between either CAA patients and AD patients or CAA patients and patients with HTN-related ICH or CAA patients and HC.

### Secondary analyses

Furthermore, we sought to meta-analyze available data on amyloid global load between CAA patients and patients with mixed (lobar and deep microbleed and/or macrobleed) pathology and between HTN-related ICH and mixed pathology.

### Study quality and assessment of publication bias

Eligible studies were subjected to quality control and bias assessment employing the Risk of Bias in Non-randomized Studies of Interventions (ROBINS-I) tool for the observational studies.^20^ The ROBINS-I tool assesses confounding, selection of participants, classification of intervention, deviations from intended intervention, missing data, measurement of outcomes, and selection of the reported result. Quality control and bias identification were performed independently by two authors (A.T. and K.M.) and any disagreements were resolved by a tie-breaking evaluator (G.T.). Moreover, GRADE approach was used to evaluate the strength of evidence.^21^

The publication bias across individual studies was evaluated graphically using funnel plots, whereas funnel plot asymmetry was assessed using Egger et al.’s linear regression test. The threshold of the statistical significance was set on *p* < 0.10.^22,23^

### Statistical approach

All statistical analyses were conducted using the R-software version 2023.06.0+421 (packages: meta and metafor).^24,25^

For the variables age and sex, we calculated the means and the prevalence rates respectively and their corresponding 95% confidence intervals (CI) to measure the effect size. With regard to mean amyloid PET uptake (continuous variable) we calculated the ratio of means, meta-analyzing patients with CAA to patients with AD, patients with CAA to patient with HTN-related ICH, and patients with CAA to HC. The variance of this ratio was calculated using the delta method and inverse variance weighting to pool ratios was also used. RoM < 1 and RoM > 1 indicates that amyloid PET uptake is lower or higher in the CAA patient population that in the comparison group respectively. This statistical approach of using fold change allows the pooling of studies using either different tracers or different methods of quantifying tracer uptake. For the qualitative interpretation of the heterogeneity, *I*^2^ > 50% and *I*^2^ > 75% indicated substantial and considerable heterogeneity, respectively.^26^ Random-effects models (DerSimonian and Laird) were used, incorporating between-study heterogeneity.^27^ A *p*-value of 0.05 or less was considered significant.

## Results

### Study selection and study characteristics

We screened 247 titles and abstracts from which 37 eligible studies were retained for full text evaluation. After careful evaluation of the available literature, 21 studies were excluded (Supplemental Table S1) resulting in selection of 16 studies that met the inclusion criteria for the present meta-analysis^19,28–42^ (PRISMA; Supplemental Figure S1 and Tables S2 and S3).

### Study quality and publication bias

The risk of bias in the included observational studies was assessed by the Risk of Bias in Non-randomized Studies of Interventions (ROBINS-I) tool^20^ and is presented in the Supplemental Figures S2 and S3. Due to the limited number of participants, control for confounders was restricted. Bias in classification of intervention, bias due to deviations from intended interventions and bias due to missing data were low. However, selection bias was observed, and information/reporting biases were moderate. According to GRADE framework, the certainty of evidence was low in the main outcomes (Supplemental Table S4).

We inspected funnel plot asymmetry and Egger et al. statistical test for outcomes involving ⩾4 cohorts (Supplemental Figures S4–S8).^22,23^ Funnel plot inspection revealed no evidence of asymmetry in included studies.

### Overall Analysis for amyloid PET

The mean age of the patients in the included studies was 72 years (95% CI: 70.3–73.7; *I*^2^ = 59%; Supplemental Figure S9) for the CAA-group, 73 years(95% CI: 71.4–74.4; *I*^2^ = 0%; Supplemental Figure S10) for the AD-group, 66 years (95% CI: 62.3–70.5; *I*^2^ = 93%; Supplemental Figure S11) for patients with HTN-related ICH, and 71 years (95% CI: 66.5–76.2; *I*^2^ = 83%; Supplemental Figure S12) for the HC. The prevalence of female sex was 46% (95% CI: 37–55; P for Cochran Q statistic = 0.02; *I*^2^ = 52%; Supplemental Figure S13) for the CAA-group, 44% (95% CI: 36–53; P for Cochran Q statistic = 0.83; *I*^2^ = 0%; Supplemental Figure S14) for the AD-group, 36% (95% CI: 26–46; P for Cochran Q statistic = 0.09; *I*^2^ = 48%; Supplemental Figure S15) for patients with HTN-related ICH, and 46% (95% CI: 25–68; P for Cochran Q statistic = 0.14; *I*^2^ = 50%; Supplemental Figure S16) for HC.

**Figure 1.**
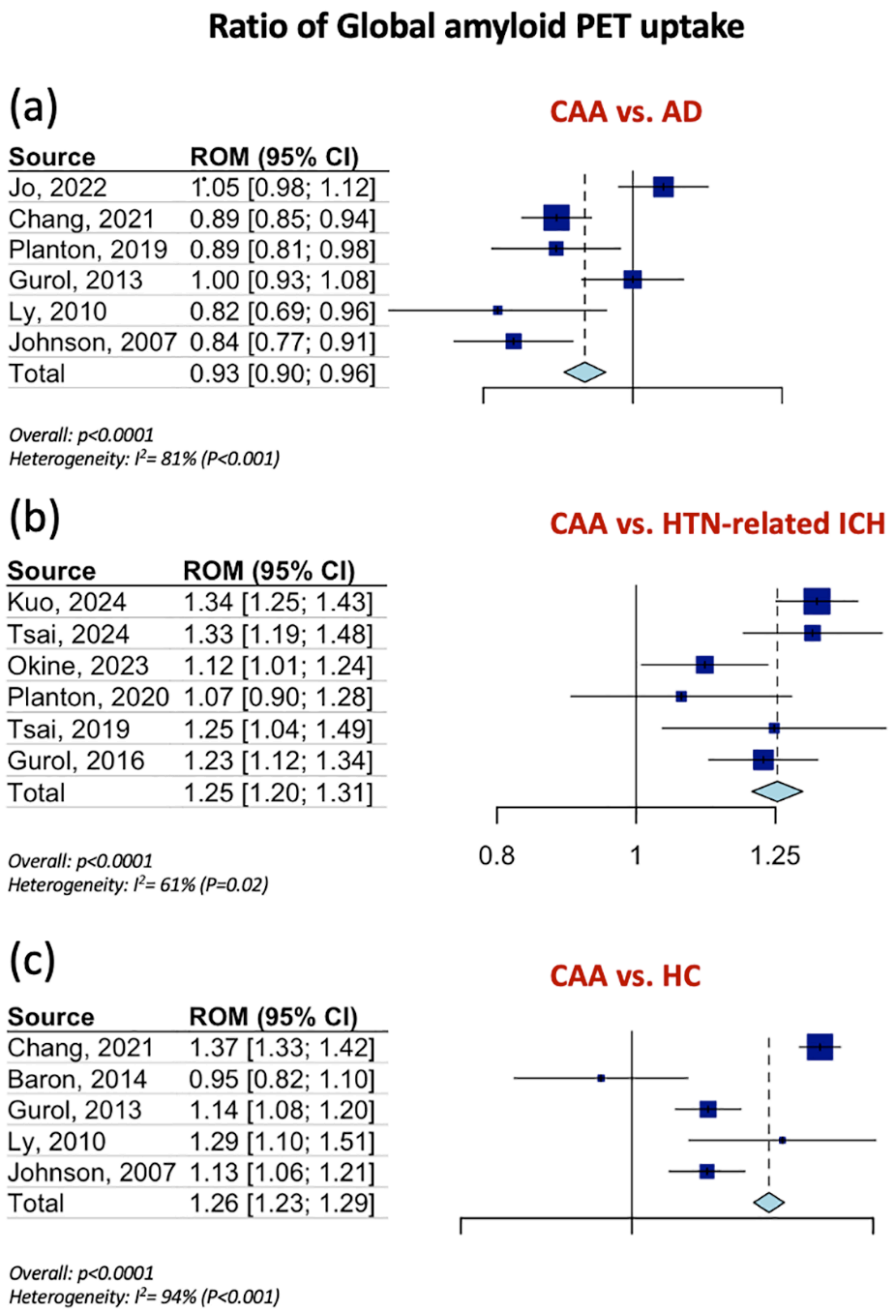
Ratios for mean global amyloid PET distribution among patients with CAA and AD (Panel a), among patients with CAA and patients with HTN-related ICH (Panel b) and among patients with CAA and HC (Panel c).

**Figure 2.**
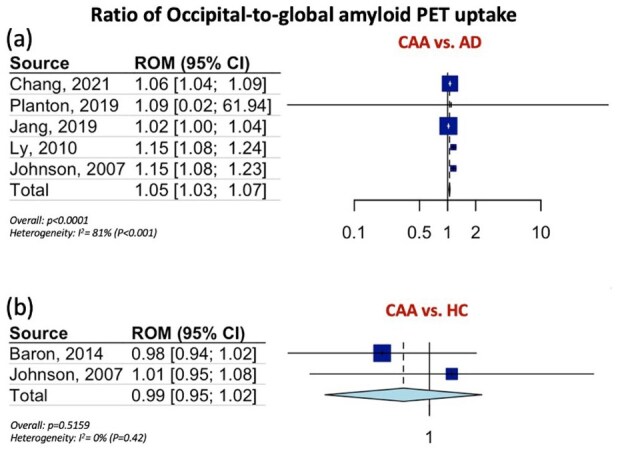
Ratios for occipital-to-global amyloid PET distribution among patients with CAA and AD (Panel a), and among patients with CAA and HC (Panel b).

**Figure 3.**
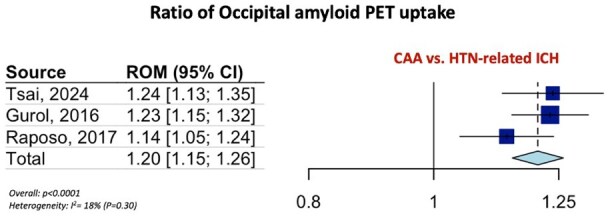
Ratio for occipital amyloid PET distribution among patients with CAA and HTN-related ICH.

Table 1 summarizes the pooled RoM for the global and regional amyloid load distribution as detected on amyloid-PET in CAA patients in comparison to AD patients, patients with HTN-related ICH and HC. In brief, global amyloid PET ratio differentiated CAA from AD [RoM: 0.93; 95% CI: 0.90–0.96; *p* < 0.0001; *I*^2^ = 81%; Figure 1(a)], HTN-related ICH [RoM: 1.25; 95% CI: 1.20–1.31; *p* < 0.0001; *I*^2^ = 61%; Figure 1(b)], and HC [RoM: 1.25; 95% CI: 1.21–1.28; *p* < 0.0001; *I*^2^ = 94%; Figure 1(c)]. Occipital-to-global ratio of amyloid-PET uptake differentiated also CAA from AD [RoM: 1.05; 95% CI: 1.03–1.07; *p* < 0.0001; *I*^2^ = 81%; Figure 2(a)], but not CAA from HC [RoM: 0.99; 95% CI: 0.95–1.02; *p* = 0.5159; *I*^2^ = 0%; Figure 2(b)]. Moreover, occipital amyloid-PET uptake [RoM: 1.20; 95% CI: 1.15–1.26; *p* < 0.0001; *I*^2^ = 18%; Figure 3] differentiated CAA from HTN-related ICH. Frontal-to-global amyloid PET uptake ratio did not differ between patients with CAA and patients with AD [RoM: 0.99; 95% CI: 0.98–1.00; *p* = 0.0799; *I*^2^ = 45%; Figure S17a] or HC [RoM: 0.99; 95% CI: 0.96–1.01; *p* = 0.2679; *I*^2^ = 0%; Figure S17b], whereas frontal amyloid-PET uptake was higher [RoM: 1.22; 95% CI: 1.13–1.32; *p* < 0.0001; *I*^2^ = 75%; Supplemental Figure S18] in CAA versus HTN-related ICH.

**Table 1.** Ratio of Mean (RoM) global or regional amyloid PET distribution comparing CAA patients versus AD patients, patients with HTN-related ICH and HC in the overall meta-analysis.

Amyloid PET distribution		RoM	95% Confidence interval	P for Cochran Q statistic	*I* ^2^
Global amyloid PET uptake	CAA/AD	0.93	0.90–0.96	<0.0001	81
	CAA/HTN	1.25	1.20–1.31	<0.0001	61
	CAA/HC	1.26	1.23–1.29	<0.0001	94
Occipital/global amyloid PET uptake	CAA/AD	1.05	1.03–1.07	<0.0001	81
	CAA/HC	0.99	0.95–1.02	0.5159	0
Occipital amyloid PET uptake	CAA/HTN	1.20	1.15–1.26	<0.0001	18
Frontal/global amyloid PET uptake	CAA/AD	0.99	0.98–1.00	0.0799	75
	CAA/HC	0.99	0.96–1.01	0.2679	0
Frontal amyloid PET uptake	CAA/HTN	1.22	1.13–1.32	<0.0001	75

AD: Alzheimer disease; CAA: cerebral amyloid angiopathy; HC: healthy controls; HTN: hypertension, PET: positron emission tomography, p-tau: phosphorylated tau, RoM: ratio of mean.

Additional analyses showed that global amyloid PET ratio differentiated CAA from mixed pathology [RoM: 1.15; 95% CI: 1.03–1.29; *p* = 0.016; *I*^2^ = 54%; Supplemental Figure S19], however failed to differentiate HTN-related ICH from mixed pathology [RoM: 1.01; 95% CI: 0.96–1.06; *p* = 0.711; *I*^2^ = 42%; Supplemental Figure S20].

## Discussion

The present systematic review and meta-analysis showed a distinct pattern of regional amyloid load distribution, detected on amyloid PET among CAA patients. CAA is characterized by a lower global amyloid PET retention compared to AD, and a higher global amyloid load compared to patients with HTN-related ICH and HC. CAA patients had higher occipital amyloid load compared to patients with HTN-associated ICH and the occipital-to-global amyloid PET uptake ratio is significantly higher in CAA compared to AD. Non-significant differences were detected with regard to frontal-to-global amyloid PET uptake ratio among CAA patients and AD patients or HC.

These findings with respect to amyloid-PET retention in CAA are in accordance with the results of a previous smaller-scale meta-analysis and different observational cohort studies.^28,29,32,33,43^ Notably, the earlier meta-analysis on this topic has included only seven studies reporting data from 106 patients with CAA, whereas the present updated meta-analysis has included data from 16 observational prospective cohort studies, which comprised 271 CAA patients, strengthening the reliability and robustness of our estimates.^43^ To strengthen the robustness of our results, we followed a pre-specified and registered in the PROSPERO database protocol of this systematic review and meta-analysis, predefining all the primary and secondary outcomes. Compared to the previous meta-analysis, the present study assessed not only the global amyloid PET ratio and the occipital-to-global amyloid PET ratio. It provided also evidence with regard to the occipital amyloid PET ratio, the frontal amyloid PET ratio and the frontal-to-global amyloid PET ratio between CAA patients and comparison subgroups.

The emerging evidence for the role of amyloid PET highlights the potential for CAA pathology detection at preliminary, probably asymptomatic stages. A multidimensional work-up, including clinical characteristics, neuroimaging/genetic findings, evidence from CSF biomarkers evaluation and amyloid PET retention may lead to a prompt and more reliable diagnosis of CAA patients, and may also contribute to the monitoring of disease progression.^3,8,9^ The significant role of CSF biomarkers has already been revealed in a recent meta-analysis, demonstrating a distinct CSF biomarker pattern, with lower Aβ40 levels in CAA compared to HC and AD, whereas tau and p-tau levels are shown to be higher in CAA compared to HC, but lower in comparison to AD patients.^9^ This diagnostic approach, and especially the established use of amyloid PET, could also serve as a basis for identifying patients eligible for novel anti-amyloid β monoclonal antibody treatments.

The value of amyloid PET use in presymptomatic CAA patients could also be supported by data from presymptomatic CAA mutation carriers.^4,44,45^ This evidence provides important insights into variable biomarkers for early disease, including also increased retention of the PET agent ^11^C-PiB on amyloid imaging. Major limitations in this approach remain the limited availability of amyloid-PET imaging and the substantial cost of this diagnostic method.

Due to the frequent overlapping underlying neuropathology, clinical and neuroimaging discrimination between CAA and AD remains a huge challenge. Interestingly, histopathological evidence reports that the vast majority of patients with AD have signs of CAA coexistence.^1^ The results of the present meta-analysis, showing lower global amyloid deposition associated with higher occipital deposition in CAA compared to AD, confirmed the findings of previous cohort studies.^28,31,33^ Notably, significant correlation between regional amyloid deposition and cortical microbleeds localization has been also described in previous studies, showing also the occipital predominance of CAA and the variable amyloid deposition at the CMB sites in CAA compared to AD and HC.^14,32,46^ In clinical practice, the occipital predominance of amyloid-PET retention among CAA patients could be used as supportive evidence combined with the more common MRI neuroimaging markers, in order to distinguish CAA from AD.

Tau PET, using the tracer flortaucipir F-18, has become widely available and well established in the AD diagnosis.^47^ However, clinical utility of tau PET imaging in the diagnosis and management of CAA patients remains sparse. Previously published studies showed increased regional tau-PET retention in regions affected predominantly by cerebral microbleeds and cortical superficial siderosis and increased tau-PET retention among patients with amnestic CAA, especially in regions susceptible to pathologic tau accumulation in AD.^29,48^ The combined use of amyloid and tau PET could also contribute to a more precise recognition of CAA pathology. Moreover, restricted evidence exists with regard to the utility of [^18^F] fluorodeoxyglucose (FDG)-PET on the differentiation of CAA from AD, which requires further investigation on larger prospective studies.^49,50^

Several methodological nuances should be considered for an accurate interpretation of the current findings. First, the moderate sample size, due to the limited data availability with regard to amyloid PET, represents the main limitation of our study. The present meta-analysis including only small, non-randomized cohort studies, imposed limitations on making any recommendations which could affect the diagnostic process and management of patients with CAA. Unfortunately, considering the limited number of included studies in association with scarce evidence of interest, a meta-regression analysis could not be pursued in order to further explore whether different neuroimaging findings (e.g. the number or the localization of CMBs, the presence of non-hemorrhagic markers, such as white matter lesions or enlarged perivascular spaces in the centrum semiovale) may significantly moderate the study outcomes. Second, all included studies used the proposed Boston criteria for the diagnosis of probable/ possible CAA diagnosis, and none of them relied on histopathological confirmation of the disease. Using only clinic-radiological criteria the possibility of overlapping CAA among patients diagnosed with AD, HTN-related ICH or even HC could not be ruled out, thus diminishing the results of our study. Nevertheless, it should be noted that the vast majority of the included patients (more than 241 out of the 271 patients) fulfilled the criteria for probable CAA, excluding possible CAA category, which has been associated with a lower accuracy to identify CAA. Third, only two amyloid tracers were investigated in the present meta-analysis, since limited data and studies exist with regard to novel tracers, such as ^18F^flutemetamol.^51^ Fourth, lack of consensus on timing of screening for CAA is another limitation of our study, since the possible effect of disease stage on PET imaging and on discrimination of AD, remains controversial. Last, there were no available data with regard to tau PET retention in CAA versus AD, which could be implemented for earlier recognition of CAA and more reliable discrimination from AD.

In conclusion, the present systematic review and meta-analysis provide evidence that amyloid load and distribution as detected on amyloid-PET may be able to distinguish CAA from AD, HTN-related ICH and HC. Future research based on larger, prospective, imaging-pathology correlation studies, is necessary to validate the results of the present systematic review and meta-analysis. This may contribute to the design and conduct of future clinical trials, with the aim to treat CAA patients at preclinical, asymptomatic stages.

## Supplementary Material

sj-docx-1-eso_23969873251349657

## Data Availability

All data needed to evaluate the conclusions in the paper are present in the main manuscript and in the supplemental material. Additional data related to this paper may be requested from the corresponding author, upon reasonable request.
