# Supplementary material for: Cerebral amyloid angiopathy and amyloid load distribution detected on amyloid–positron emission tomography: A systematic review and meta-analysis
Source: Eur Stroke J. 2026 Jan 1;11(1):23969873251349657. doi: 10.1093/esj/23969873251349657 (PMC12866260; doi:10.1093/esj/23969873251349657)
Supplement: sj-docx-1-eso_23969873251349657 [file sj-docx-1-eso_23969873251349657.docx]

**SUPPLEMENTAL MATERIAL**

**TITLE:** **Cerebral amyloid angiopathy and amyloid load distribution detected on amyloid–positron emission tomography: A systematic review and meta-analysis**

**Complete search algorithm used in MEDLINE search**

("positron emission tomography"[MeSH Terms] OR ("positron emission"[All Fields] AND "tomography"[All Fields]) OR "positron emission tomography"[All Fields] OR ("positron"[All Fields] AND "emission"[All Fields] AND "tomography"[All Fields]) OR "positron emission tomography"[All Fields]) AND ("cerebral amyloid angiopathy"[MeSH Terms] OR ("cerebral"[All Fields] AND "amyloid"[All Fields] AND "angiopathy"[All Fields]) OR "cerebral amyloid angiopathy"[All Fields])

**Complete search algorithm used in SCOPUS search**

(TITLE-ABS-KEY (positron AND emission AND tomography) AND TITLE-ABS-KEY (cerebral AND amyloid AND angiopathy))

**Supplementary Tables:**

- **Supplementary Table S1.:** Excluded Studies with Reasons for Exclusion
- **Supplementary Table S2.:** Study Design and cohort selection of included studies in our meta-analysis
- **Supplementary Table S3.:** Basic characteristics of included studies in our meta-analysis
- **Supplementary Table S4.:** Summary of GRADE Evidence Profile

**Supplementary Figures:**

- **Supplementary Figure S1.:** PRISMA flowchart diagram presenting the selection of eligible studies.
- **Supplementary Figure S2.:** Traffic Light Plot presenting the quality assessment of included observational studies using the Risk of Bias In Non-randomized Studies of Interventions (ROBINS-I) tool.
- **Supplementary Figure S3.:** Traffic Light Plot presenting the quality assessment of included observational studies using the Risk of Bias In Non-randomized Studies of Interventions (ROBINS-I) tool.
- **Supplementary Figures S4-S8:** Funnel plots.
- **Supplementary Figures S9-S18:** Forest Plots.

**References used in the Supplement.**

**TABLES**

**Supplementary Table S1.:** Excluded Studies with Reasons for Exclusion

| **Study Name (PMID)** | **Reason for Exclusion** |
| --- | --- |
| Pyun, et al; 2024 (39044515) | No specific data on CAA patients; no comparative population |
| Romoli, et al; 2024 | No specific data on CAA patients |
| Phuah, et al; 2022 (36123127) | No specific data on CAA patients |
| Michiels, et al; 2022 (35853346) | Available data only on CAA patients; no comparative population |
| Finze, et al; 2022 (35185518) | Available data only on CAA patients; no comparative population |
| Gokcal, et al; 2021 (35086372) | Available data only on CAA patients; no comparative population |
| Schoemaker, et al; 2021 (33627498) | Available data only on CAA patients; no comparative population |
| Zhang, et al; 2021 (34777194) | Available data only from [^68^Ga]Ga-p14-032 PET |
| Kim, et al; 2021 (34724019) | No data on amyloid load distribution detected on amyloid-PET |
| McCarter et al; 2021 (34504022) | No specific data on patients diagnosed with CAA. |
| Bergeret et al; 2021 (33611942) | Available data only from ^18^F-FDG PET |
| Bergeret et al; 2021 (33460498) | Available data only from ^18^F-FDG PET |
| Alakbarzade, et al; 2020 (33644295) | No data on amyloid load distribution detected on amyloid-PET |
| Banerjee, et al; 2020 (32176643) | No data on amyloid load distribution detected on amyloid-PET |
| Jung, et al; 2020 (33198580) | No data on amyloid load distribution detected on amyloid-PET |
| Jung, et al; 2020 (33139780) | No data on amyloid load distribution detected on amyloid-PET |
| Low, et al; 2020 (32917821) | No data on CAA patients |
| Tsai, et al; 2020 (31726962) | No specific data on CAA patients |
| Graff-Radford, et al; 2018 (30568001) | No data on CAA patients |
| Renard, et al; 2017 (29254099) | No data on CAA patients |
| Charidimou, et al; 2017 (28855406) | Systematic review and meta-analysis |
| Charidimou, et al; 2017 (29070646) | Systematic review and meta-analysis |

**Supplementary Table S2.:** Study Design and cohort selection of included studies in our meta-analysis

| **First Author** | **Year** | **Stroke Center/Clinic; Country** | **Data Collection Interval** | **Study Design prospective** | **Tracer used** | **SUVR vs. DVR** | **CAA diagnostic criteria** | **Alzheimer Disease diagnostic criteria** | **Hypertension-related ICH - definition** | **Healthy Controls definition** |
| --- | --- | --- | --- | --- | --- | --- | --- | --- | --- | --- |
| Kuo, et al^1^ | 2024 | National  Taiwan University Hospital (NTUH) | 2014-2022 | Observational, prospective cohort study | ^11^C-PIB | SUVR | Boston criteria  1.5 | N.A. | Arteriosclerotic pathologies  in the deep-seated vessels that stem directly from the large vessels as arterial perforators | N.A. |
| Tsai, et al^2^ | 2024 | National  Taiwan University Hospital (NTUH) | 2019 onwards | Observational, prospective cohort study | ^11^C-PIB | SUVR | Boston criteria  2.0 | N.A. | Patients with haemorrhage/CMBs exclusively located in deep brain regions (basal ganglia, thalamus or pons) or patients with a combination of mixed lobar and deep bleeds | N.A. |
| Okine, et al^3^ | 2023 | ARIC-PET study (Atherosclerosis Risk  in Communities - Positron Emission Tomography)  -  3 ARIC sites  (Jackson, MS; Washington County, MD; and Forsyth County,  NC) | 2012 - 2014 | Observational, prospective cohort study | ^18^F-florbetapir | SUVR | N.A. | N.A. | N.A. | N.A. |
| Jo, et al^4^ | 2022 | Asan Medical Center, Seoul | 2015 - 2020 | Observational, prospective cohort study | ^18^F-florbetapir | SUVR | modified Boston criteria | National Institute on Aging and the  Alzheimer’s Association criteria | N.A. | N.A. |
| Chang, et al^5^ | 2021 | Department of Neurologic Medicine in Chinese PLA General  Hospital, China | 2017 - 2019 | Observational, prospective cohort study | ^11^C-PIB | SUVR | Boston Criteria | NINCDS–  ADRDA | N.A. | Without cognition impairment |
| Planton, et al^6^ | 2020 | Toulouse, France | N.A. | Observational, prospective cohort study | ^18^F-florbetapir | SUVR | modified Boston Criteria | N.A. | Non-demented patients with acute  symptomatic deep ICH | N.A. |
| Planton, et al^7^ | 2019 | Toulouse, France | N.A. | Observational, prospective cohort study | ^18^F-florbetapir | SUVR | modified Boston Criteria | NINCDS-ADRDA | N.A. | N.A. |
| Jang, et al^8^ | 2019 | Samsung Medical Center, Korea | 2015-2016 | Observational, prospective cohort study | ^11^C-PIB or  ^18^F-florbetapir | SUVR | modified Boston Criteria | National Institute on Aging and the Alzheimer’s Association  criteria | N.A. | N.A. |
| Tsai, et al^9^ | 2019 | National  Taiwan University Hospital (NTUH) | 2014 -2017 | Observational, prospective cohort study | ^11^C-PIB | SUVR | Boston criteria | N.A. | Patients with ICH in the basal  ganglia (BG), thalamus, or infratentorial region (deep locations)  with or without deep CMBs but no lobar CMB | N.A. |
| Raposo, et al^10^ | 2017 | Toulouse, France | 2012 -2016 | Observational, prospective cohort study | ^18^F-florbetapir | SUVR | modified Boston criteria | N.A. | Patients with acute primary symptomatic deep ICH | N.A. |
| Tsai, et al^11^ | 2017 | National  Taiwan University Hospital (NTUH) | 2014 -2016 | Observational, prospective cohort study | ^11^C-PIB | SUVR | modified Boston criteria | N.A. | Hypertensive ICH is usually located in the deep region  -  Clinical diagnosis  of ICH was based on the SMASH-U criteria ^18^ | N.A. |
| Gurol, et al^12^ | 2016 | Massachusetts General Hospital, Boston | N.A. | Observational, prospective cohort study | ^18^F-florbetapir | SUVR | Boston Criteria | N.A. | Patients who had  deep hypertensive ICH (HTN-ICH) | N.A. |
| Baron, et al^13^ | 2014 | Cambridge, United Kingdom | N.A. | Observational, prospective cohort study | ^11^C-PIB | DVR | Boston Criteria | N.A. | N.A. | No memory or cognitive complaints and with normal  MMSE results |
| Gurol, et al^14^ | 2013 | Massachusetts General Hospital, Boston | 2005 - 2011 | Observational, prospective cohort study | ^11^C-PIB | DVR | Boston Criteria | National Institute of Neurological and Communicative  Disorders and Stroke–Alzheimer’s Disease and  Related Disorders Association criteria | N.A. | Healthy elderly subjects without any lobar microbleeds on  T2*-weighted MRI. Healthy elderly subjects were excluded for diabetes mellitus or for  requiring > 1 medication for hypertension or hyperlipidemia. |
| Ly, et al^15^ | 2010 | Australia | 2005 - 2007 | Observational, prospective cohort study | ^11^C-PIB | DVR | Boston Criteria | National Institute  of Neurological and Communicative Disorders and Stroke–  Alzheimer’s Disease and Related Disorders Association criteria | N.A. | Aged-matched normal controls |
| Johnson, et al^16^ | 2007 | Massachusetts General Hospital, Boston | N.A. | Observational, prospective cohort study | ^11^C-PIB | SUVR | Boston Criteria | National Institute of Neurological and Communication  Disorders-Alzheimer’s Disease and Related Disorders  Association criteria | N.A. | Older normal control subjects |
| **Abbreviations:** 11C-PIB: (11)C-labeled Pittsburgh Compound-B, AD: Alzheimer Disease, CAA: Cerebral Amyloid Angiopathy, DVR: distribution volume ratio, MMSE: Mini-Mental State Examination, MRI: Magnetic Resonance Imaging, N.A.: not available, NINCDS–ADRDA: National Institute of Neurological and Communicative Disorders and Stroke and the AD and Related Disorders Association criteria, SUVR: Standardized uptake value ratio | | | | | | | | | | |

**Supplementary Table S3.:** Basic characteristics of included studies in our meta-analysis

| **First Author** | **Year** | **Total CAA Patients, n** | **Mean age, years (sd)** | **Female, n, %** | **Definite /Probable/ Possible Diagnosis, n** | **Total AD Patients, n** | **Mean age, years (sd)** | **Female, n, %** | **Total HTN Patients, n** | **Mean age, years (sd)** | **Female, n, %** | **Total Controls, n** | **Mean age, years (sd)** | **Female, n, %** |
| --- | --- | --- | --- | --- | --- | --- | --- | --- | --- | --- | --- | --- | --- | --- |
| Kuo, et al^1^ | 2024 | 30 | 74,7 (9.4) | 18 | N.A. | N.A. | N.A. | N.A. | 61 | 63.0 (11.6) | 16 | N.A. | N.A. | N.A. |
| Tsai, et al^2^ | 2024 | 31 | 72.9 (7.5) | 17 | 0/31/0 | N.A. | N.A. | N.A. | 27 | 67.3 (10.0) | 9 | N.A. | N.A. | N.A. |
| Okine, et al^3^ | 2023 | 16 | 74,7 (4.5) | 10 | N.A. | N.A. | N.A. | N.A. | 44 | 74.3 (5.1) | 24 | N.A. | N.A. | N.A. |
| Jo, et al^4^ | 2022 | 30 | 72,2 (7.6) | 16 | 0/30/0 | 30 | 71.5 (7.6) | 16 | N.A. | N.A. | N.A. | N.A. | N.A. | N.A. |
| Chang, et al^5^ | 2021 | 9 | 78,7 23.6) | 2 | 0/9/0 | 15 | 73.7 (28.6) | 6 | N.A. | N.A. | N.A. | 15 | 78.3 (15.5) | 4 |
| Planton, et al^6^ | 2020 | 18 | 68,8 (15.0) | 9 | 0/15/3 | N.A. | N.A. | N.A. | 18 | 64.3 (10.5) | 5 | N.A. | N.A. | N.A. |
| Planton, et al^7^ | 2019 | 15 | 68.5 (15.1) | 5 | 0/15/0 | 20 | 72.6 (8.2) | 9 | N.A. | N.A. | N.A. | N.A. | N.A. | N.A. |
| Jang, et al^8^ | 2019 | 43 | 74,4 (8.1) | 23 | 0/43/0 | 129 | 67.1 (10.2) | 71 | N.A. | N.A. | N.A. | N.A. | N.A. | N.A. |
| Tsai, et al^9^ | 2019 | 13 | 73,3 (11.9) | 8 | 0/13/0 | N.A. | N.A. | N.A. | 21 | 60.1 (14.5) | 8 | N.A. | N.A. | N.A. |
| Raposo, et al^10^ | 2017 | 15 | 66.7 (12.0) | 6 | 0/15/0 | N.A. | N.A. | N.A. | 18 | 63.1 (11.0) | 5 | N.A. | N.A. | N.A. |
| Tsai, et al^11^ | 2017 | 8 | 79.9 (12.5) | N.A. | N.A. | N.A. | N.A. | N.A. | 11 | 58.5 (14.0) | NA | N.A. | N.A. | N.A. |
| Gurol, et al^12^ | 2016 | 10 | 66,9 (6.0) | 3 | 0/10/0 | N.A. | N.A. | N.A. | 9 | 67.1 (7.9) | 3 | N.A. | N.A. | N.A. |
| Baron, et al^13^ | 2014 | 11 | 70,0 (7.0) | 2 | 0/11/0 | N.A. | N.A. | N.A. | N.A. | N.A. | N.A. | 9 | 65.0 (5.0) | 5 |
| Gurol, et al^14^ | 2013 | 42 | 68,0 (10.0) | 11 | 0/42/0 | 43 | 74.0 (7.4) | 17 | N.A. | N.A. | N.A. | N.A. | N.A. | N.A. |
| Ly, et al^15^ | 2010 | 12 | 73,9 (10.3) | N.A. | 0/8/4 | 13 | 73.8 (11.7) | N.A. | N.A. | N.A. | N.A. | 22 | 71.8 (6.6) | N.A. |
| Johnson, et al^16^ | 2007 | 6 | 68,5 (11.4) | 3 | 0/6/0 | 9 | 71.4 (10.9) | 4 | N.A. | N.A. | N.A. | 15 | 72.7 (6.4) | 9 |
| **Abbreviations:** AD: Alzheimer Disease, CAA: Cerebral Amyloid Angiopathy, HTN: Patients with Hypertensive atherosclerosis, N.A.: not available | | | | | | | | | | | | | | |

**Supplementary Table S4.:** Summary of GRADE Evidence Profile

| **Certainty assessment** | | | | | | | **Effect** | **Certainty** | **Importance** |  |
| --- | --- | --- | --- | --- | --- | --- | --- | --- | --- | --- |
| **№ of studies** | **Study design** | **Risk of bias** | **Inconsistency** | **Indirectness** | **Imprecision** | **Other considerations** | **Ratio of Means (RoM) (95% CI)** |  |  |  |
| **Global Amyloid PET uptake in CAA vs. AD** | | | | | | | | | | |
| 6 | Non-randomised studies | serious | serious | not serious | not serious | none | **RoM: 0.93** (0.90 to 0.96) | ⨁⨁◯◯ Low | IMPORTANT |  |
| **Global Amyloid PET uptake in CAA vs. HTN** | | | | | | | | | | |
| 6 | Non-randomised studies | serious | serious | not serious | not serious | none | **RoM: 1.25** (1.20 to 1.31) | ⨁⨁◯◯ Low | IMPORTANT |  |
| **Global Amyloid PET uptake in CAA vs. HC** | | | | | | | | | | |
| 5 | Non-randomised studies | serious | serious | not serious | not serious | none | **RoM: 1.26** (1.23 to 1.29) | ⨁⨁◯◯ Low | IMPORTANT |  |
| **Occipital to global amyloid PET uptake in CAA vs. AD** | | | | | | | | | | |
| 5 | Non-randomised studies | serious | serious | not serious | not serious | none | **RoM: 1.05** (1.03 to 1.07) | ⨁⨁◯◯ Low | IMPORTANT |  |
| **Occipital to global amyloid PET uptake in CAA vs. HC** | | | | | | | | | | |
| 2 | Non-randomised studies | serious | not serious | not serious | not serious | none | **RoM:0.99** (0.95 to 1.02) | ⨁◯◯◯ Very low | OF LIMITED IMPORTANCE |  |
| **Occipital Amyloid PET uptake in CAA vs. HTN** | | | | | | | | | | |
| 3 | Non-randomised studies | serious | not serious | not serious | not serious | none | **RoM: 1.20** (1.15 to 1.26) | ⨁⨁◯◯ Low | IMPORTANT |  |
| **Frontal to global amyloid PET uptake in CAA vs. AD** | | | | | | | | | | |
| 5 | Non-randomised studies | serious | serious | not serious | not serious | none | **RoM: 0.99** (0.98 to 1.00) | ⨁⨁◯◯ Low | OF LIMITED IMPORTANCE |  |
| **Frontal to global amyloid PET uptake in CAA vs. HC** | | | | | | | | | | |
| 2 | Non-randomised studies | serious | not serious | not serious | not serious | none | **RoM: 0.99** (0.96 to 1.01) | ⨁◯◯◯ Very low | OF LIMITED IMPORTANCE |  |
| **Frontal amyloid PET uptake in CAA vs. HTN** | | | | | | | | | | |
| 2 | Non-randomised studies | serious | serious | not serious | not serious | none | **RoM: 1.22**  (1.13 to 1.32) | ⨁◯◯◯ Very low | OF LIMITED IMPORTANCE |  |

CI: confidence interval; RoM: Ratio of Means

**Supplemental Figures**

**Figure S1.:** PRISMA flowchart diagram presenting the selection of eligible studies.


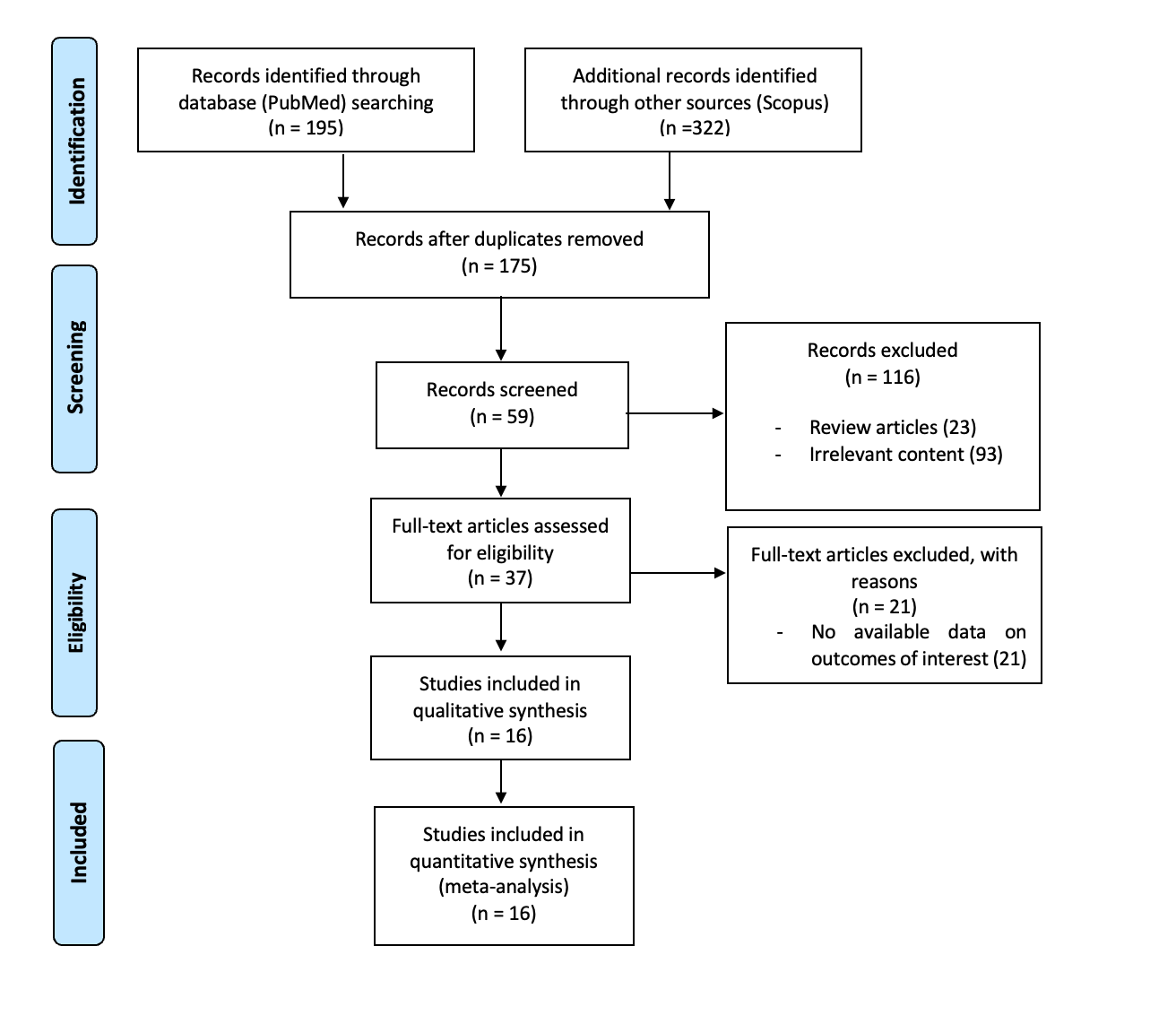


**Figure S2.:** Traffic Light Plot presenting the quality assessment of included observational studies using the Risk Of Bias In Non-randomized Studies of Interventions (ROBINS-I) tool.^19^

^
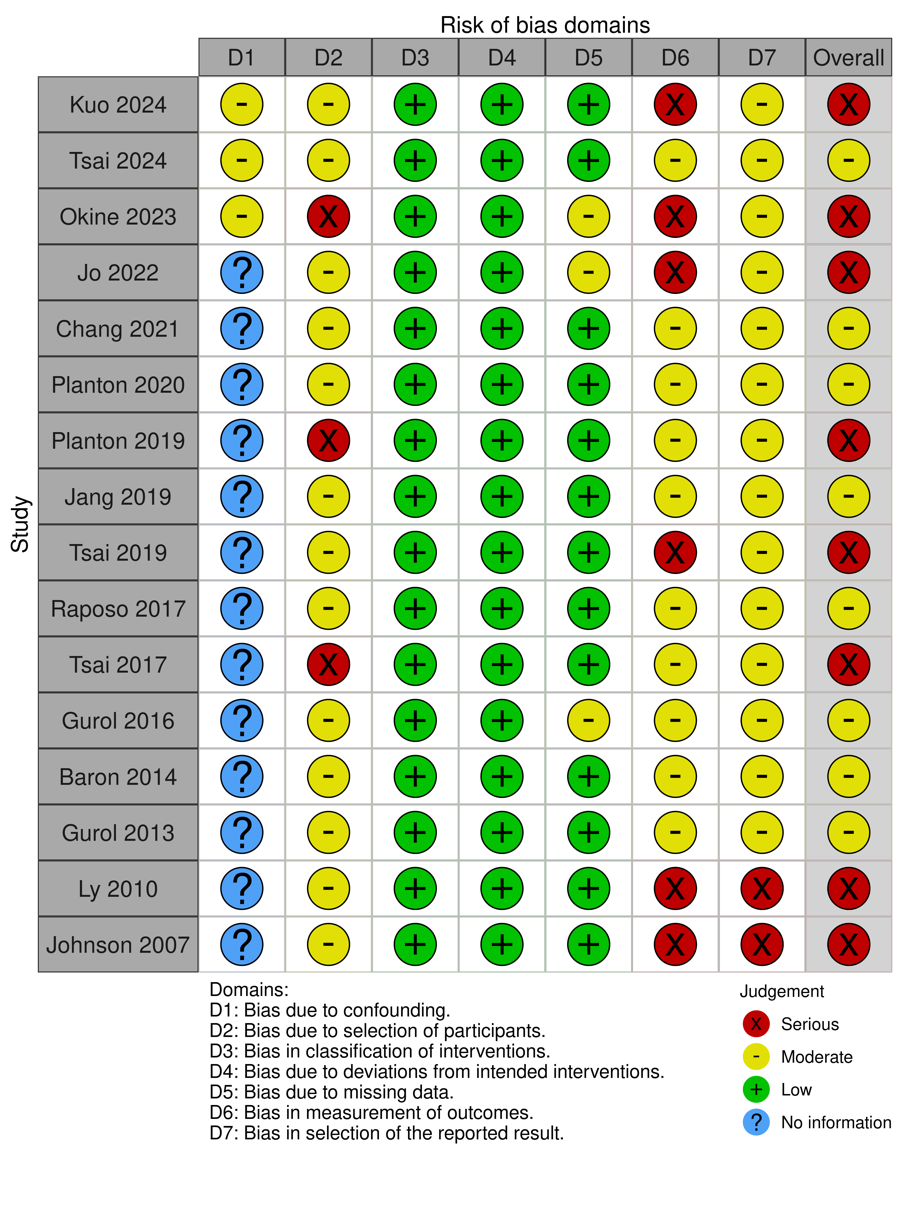
^

**Figure S3.:** Traffic Light Plot presenting the quality assessment of included observational studies using the Risk Of Bias In Non-randomized Studies of Interventions (ROBINS-I) tool.^19^


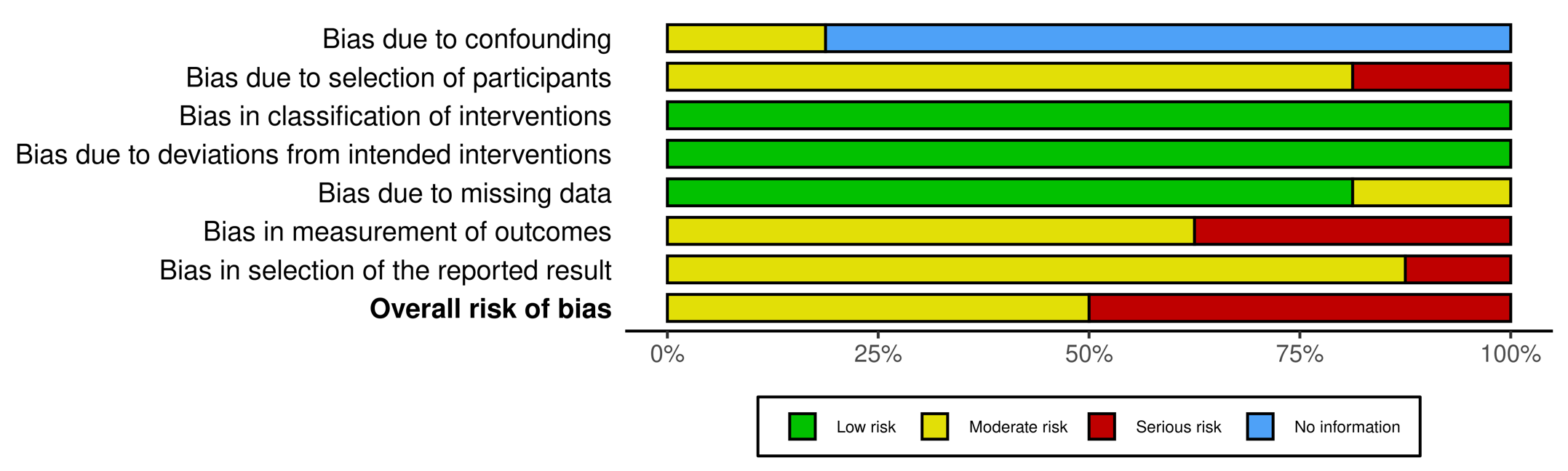


**Figure S4:** Funnel Plots for the assessment of Publication Bias Among Trials Reporting the mean age among patients with Cerebral amyloid Angiopathy (p-value for Egger’s test: 0.4133; figure A), among patients with Alzheimer disease (p-value for Egger’s test: 0.7667; figure B), among patients with hypertensive arteriosclerosis/ deep hypertensive intracerebral hemorrhage (p-value for Egger’s test: 0.0408; figure C) and among Control participants (p-value for Egger’s test: 0.9071; figure D)


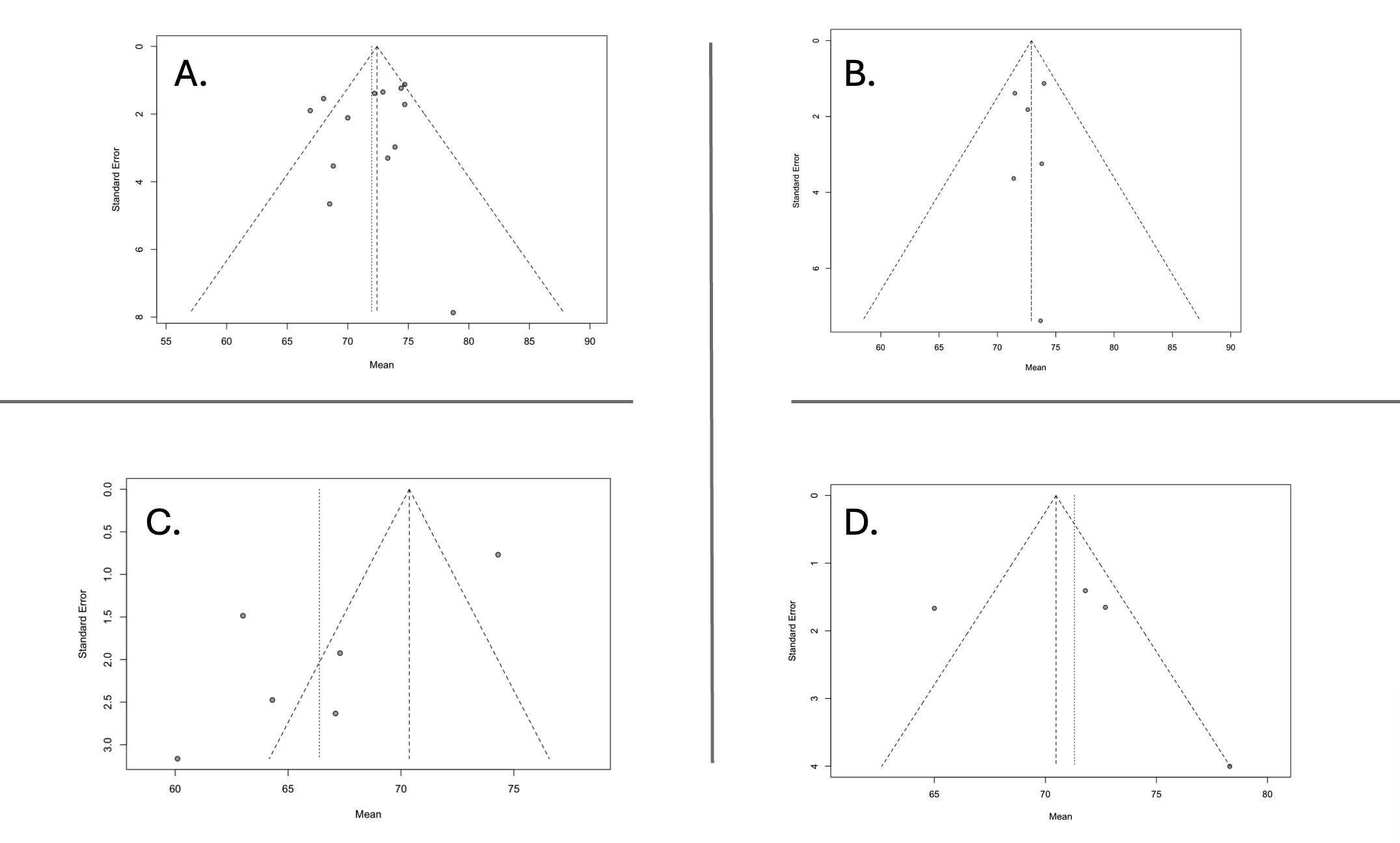


**Figure S5.:** Funnel Plots for the assessment of Publication Bias Among Trials Reporting the female sex proportion among patients with Cerebral Amyloid Angiopathy (p-value for Egger’s test: 0.6014; figure A), among patients with Alzheimer disease (p-value for Egger’s test: 0.9331; figure B), and among patients with hypertensive arteriosclerosis/ deep hypertensive intracerebral hemorrhage (p-value for Egger’s test: 0.9633; figure C).

**
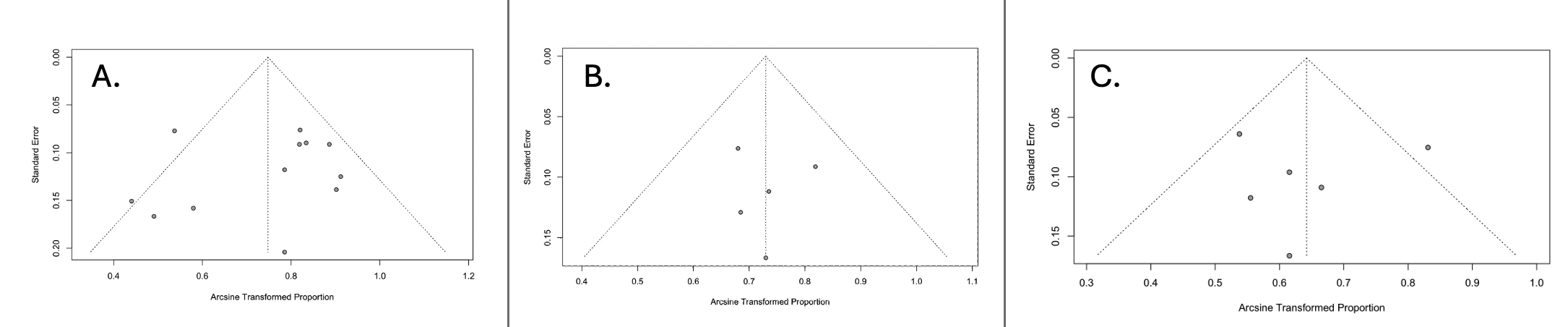
**

**Figure S6.:** Funnel Plots for the assessment of Publication Bias Among Trials Reporting the ratio of Global Amyloid PET load between patients with CAA and Alzheimer Disease (p-value for Egger’s test: 0.5866; Figure A), between patients with CAA and patients with hypertensive arteriosclerosis/ deep hypertensive intracerebral hemorrhage (p-value for Egger’s test: 0.2573; Figure B) and between patients with CAA and healthy controls (p-value for Egger’s test: 0.2330; Figure C).


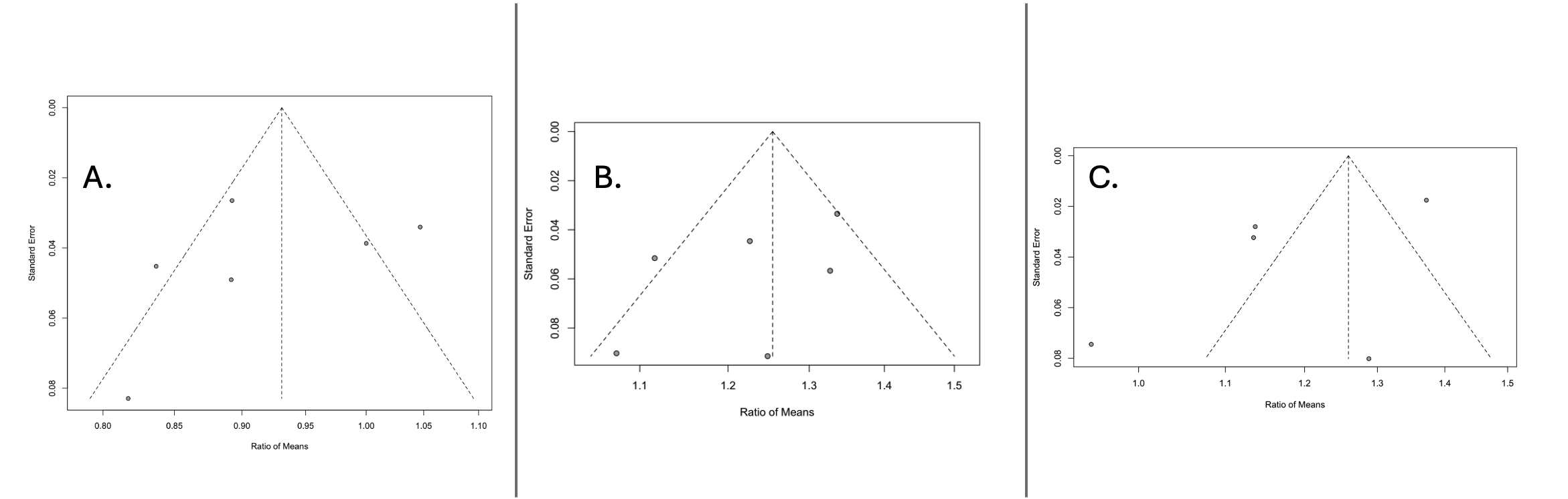


**Figure S7.:** Funnel Plot for the assessment of Publication Bias Among Trials Reporting the ratio of Occipital-to-global Amyloid PET load between patients with CAA and Alzheimer Disease (p-value for Egger’s test: 0.2727).

**
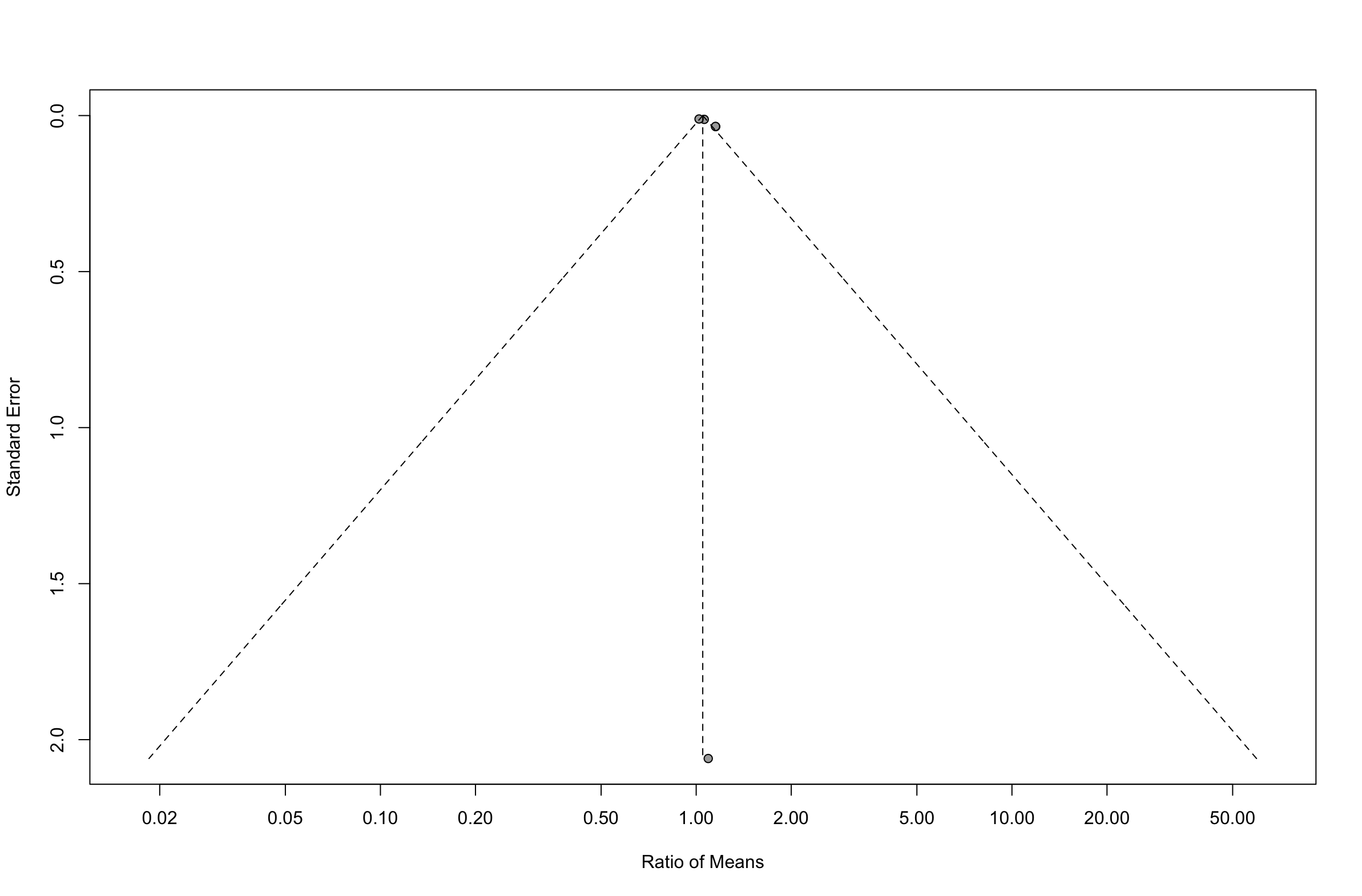
**

**Figure S8.:** Funnel Plot for the assessment of Publication Bias Among Trials Reporting the ratio of Frontal-to-global Amyloid PET load between patients with CAA and Alzheimer Disease (p-value for Egger’s test: 0.0971).


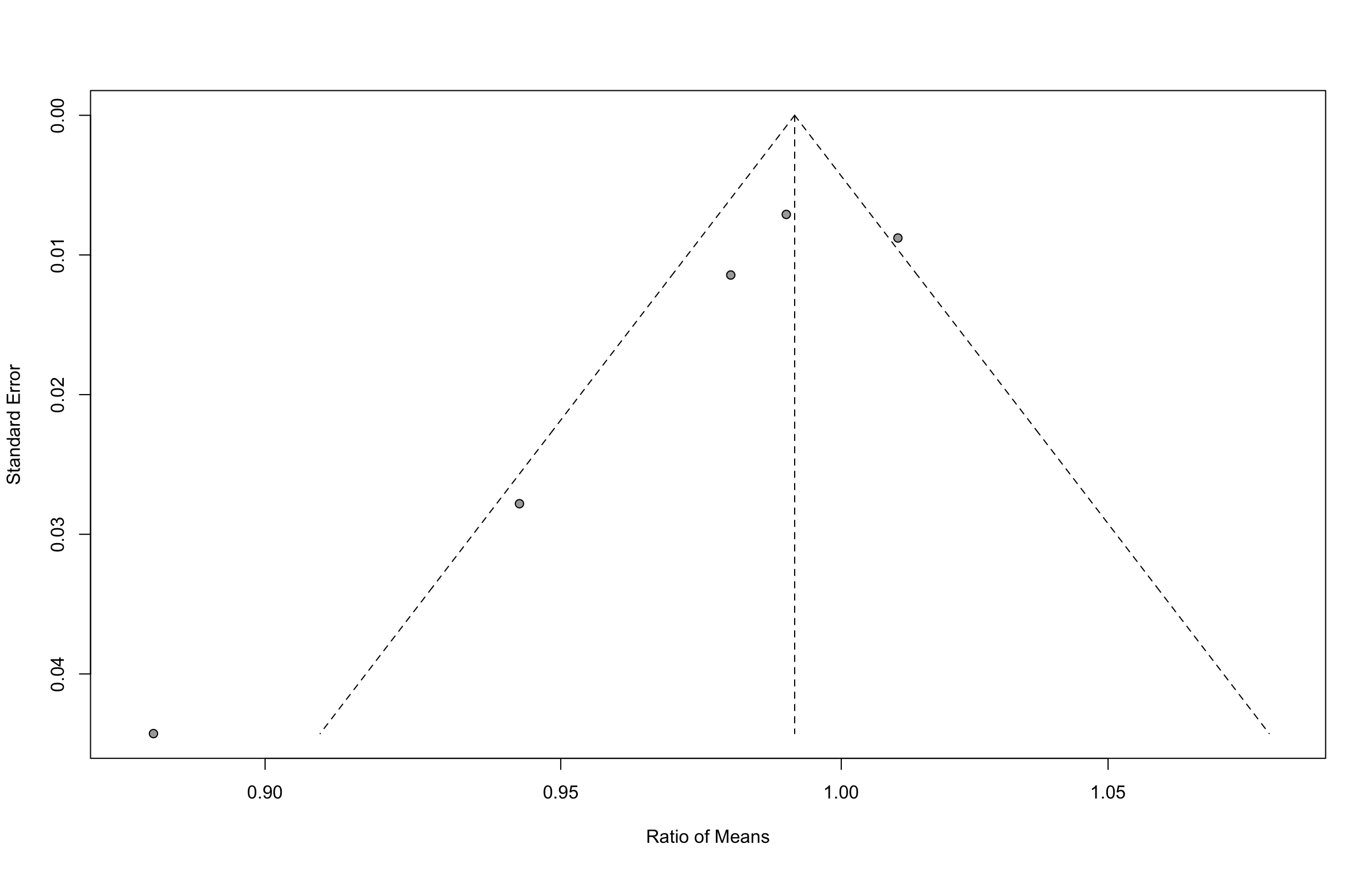


**Figure S9.:** Forest Plot presenting the mean age among patients with Cerebral Amyloid Angiopathy

**
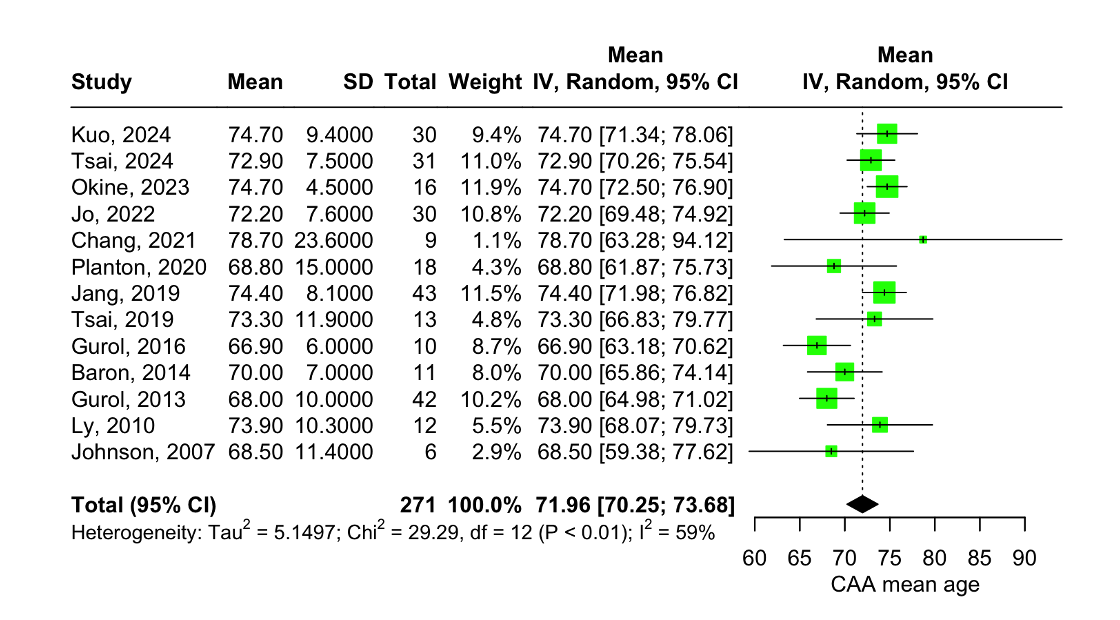
**

**Figure S10.:** Forest Plot presenting the mean age among patients with Alzheimer Disease

**
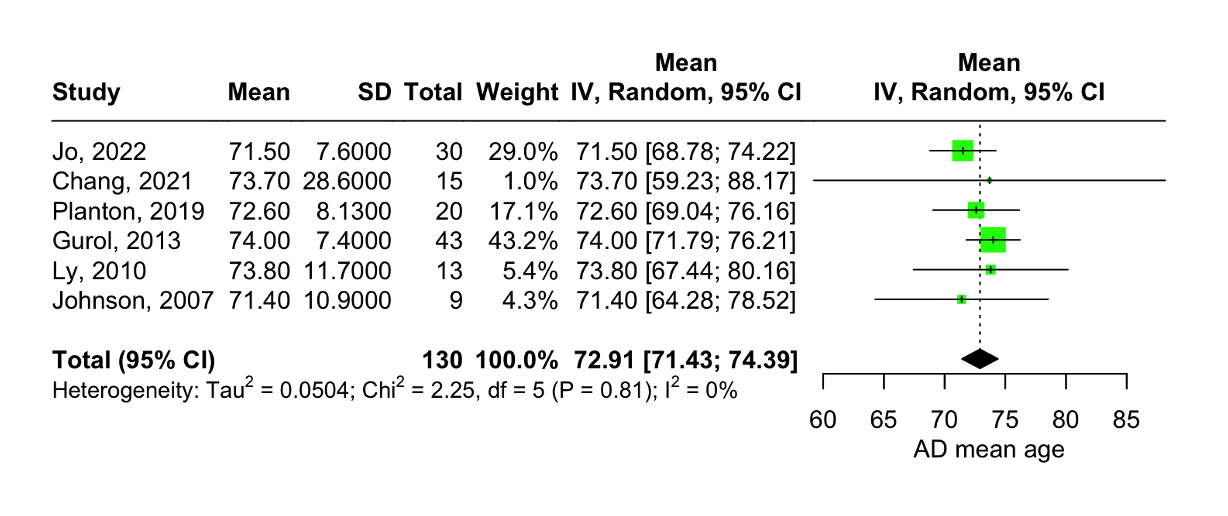
**

**Figure S11.:** Forest Plot presenting the mean age among patients with hypertensive arteriosclerosis/ deep hypertensive intracerebral hemorrhage.


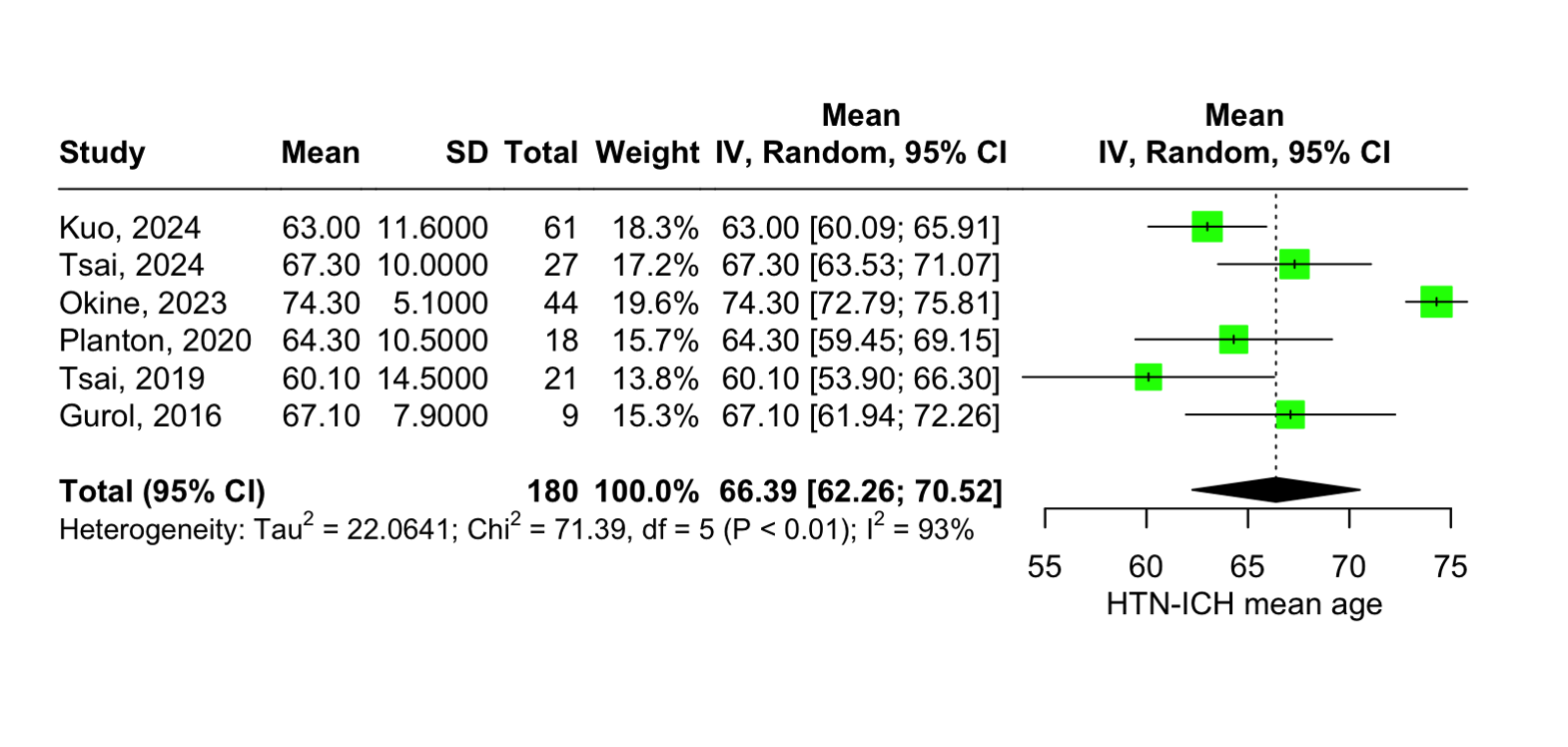


**Figure S12.:** Forest Plot presenting the mean age among Healthy Control Participants


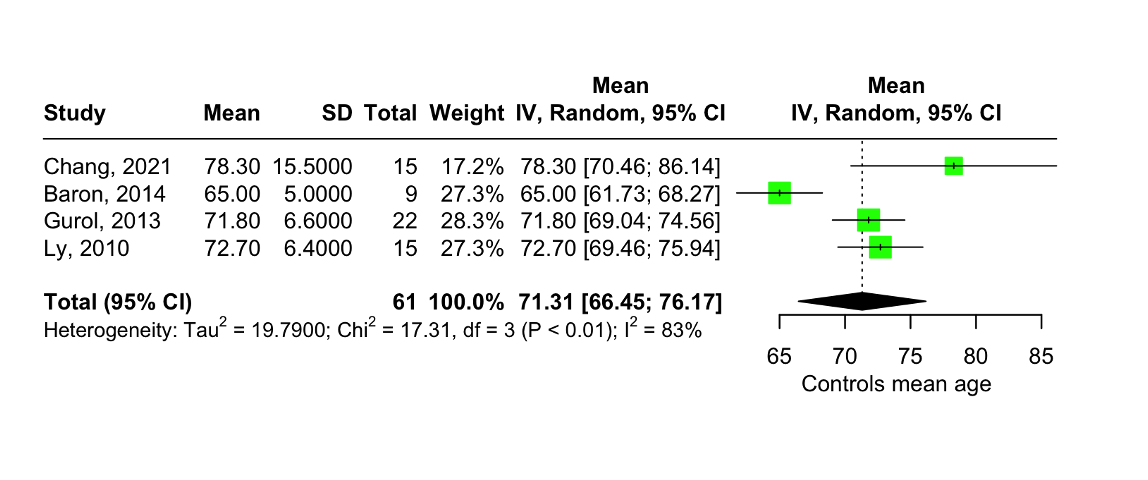


**Figure S13.:** Forest Plot presenting the pooled rate of female sex among patients with Cerebral Amyloid Angiopathy

**
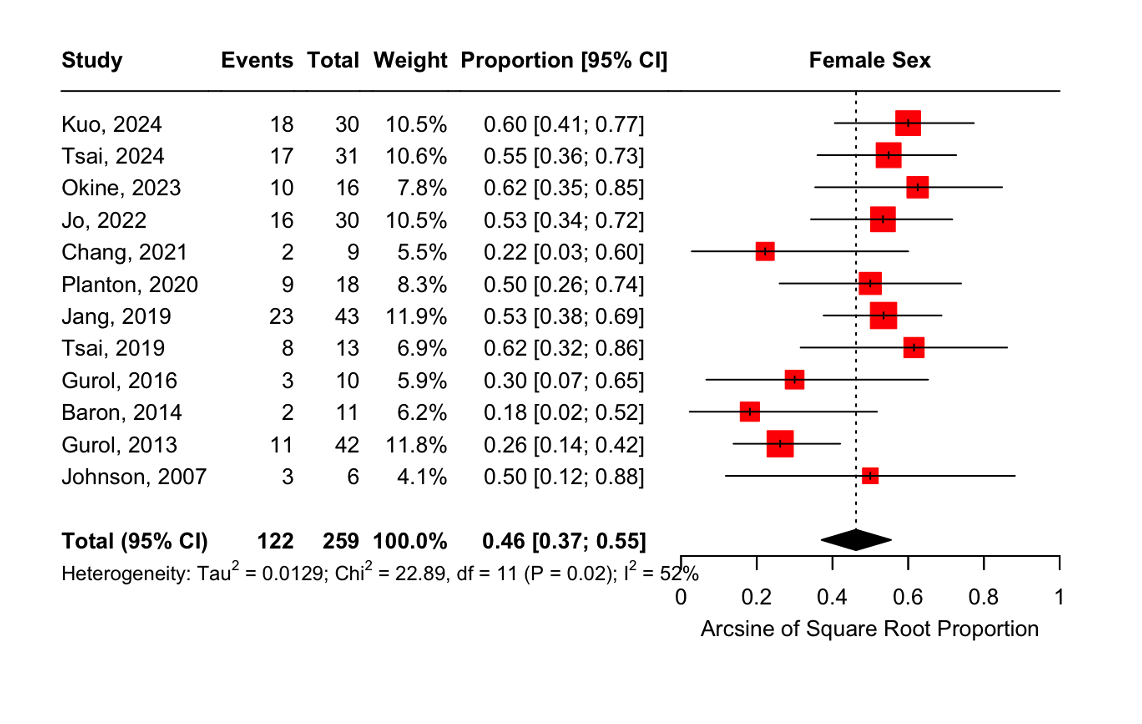
**

**Figure S14.:** Forest Plot presenting the pooled rate of female sex among patients with Alzheimer Disease


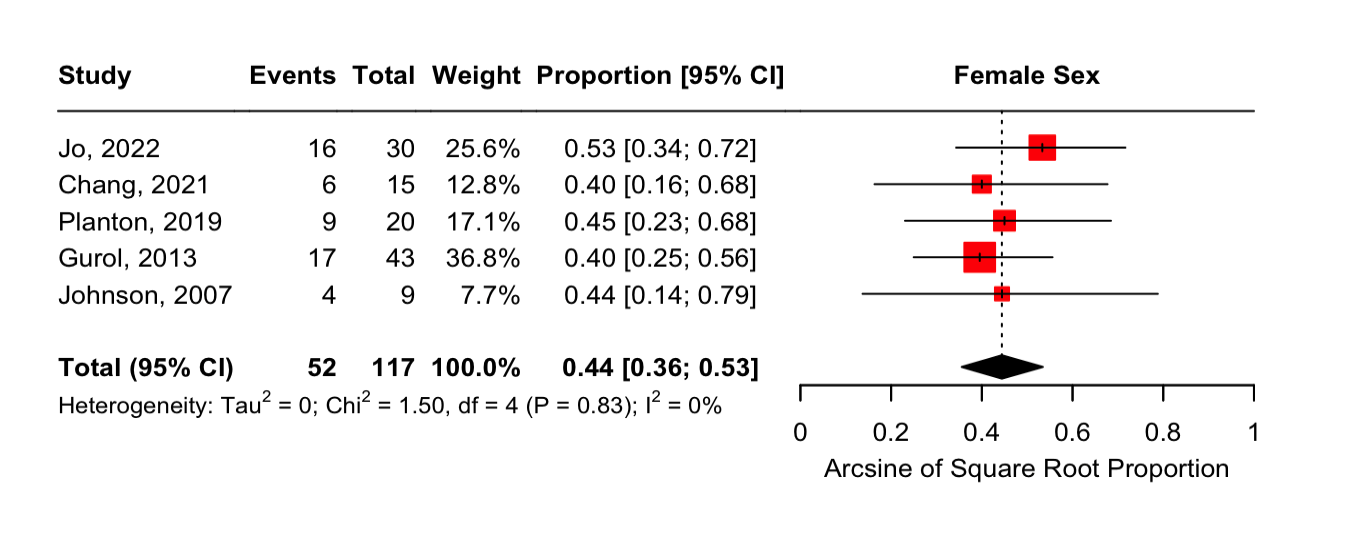


**Figure S15.:** Forest Plot presenting the pooled rate of female sex among patients with hypertension related intracerebral hemorrhage.


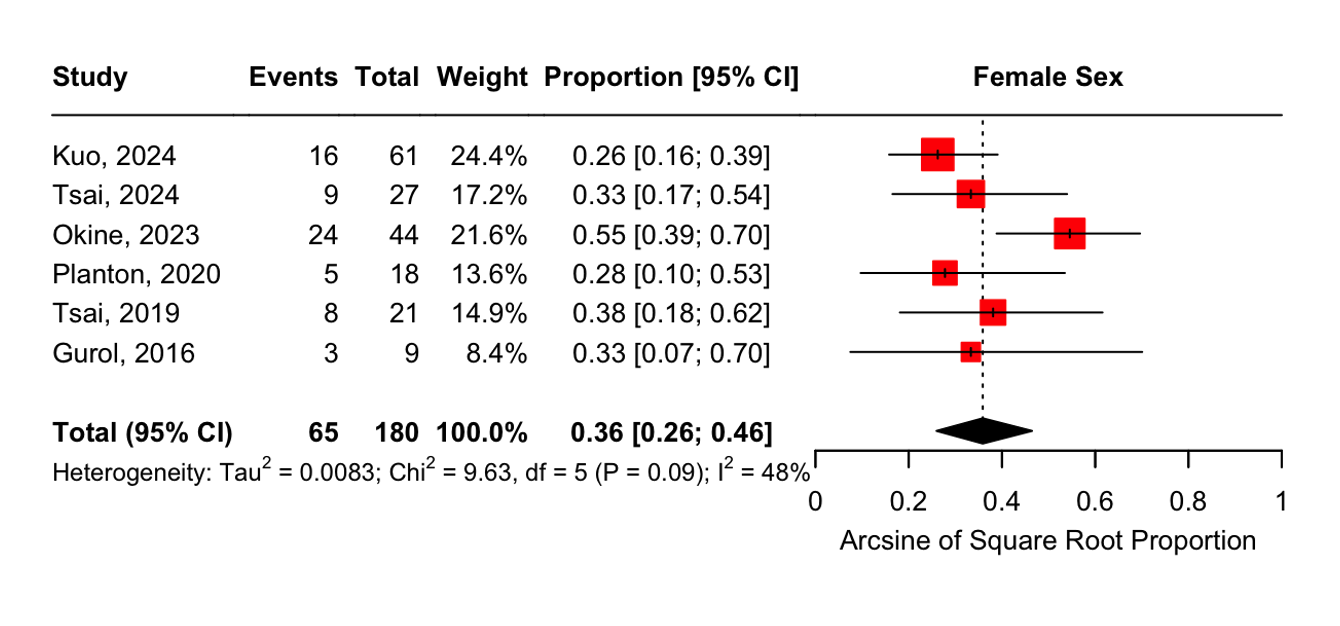


**Figure S16.:** Forest Plot presenting the pooled rate of female sex among Control Participants

**
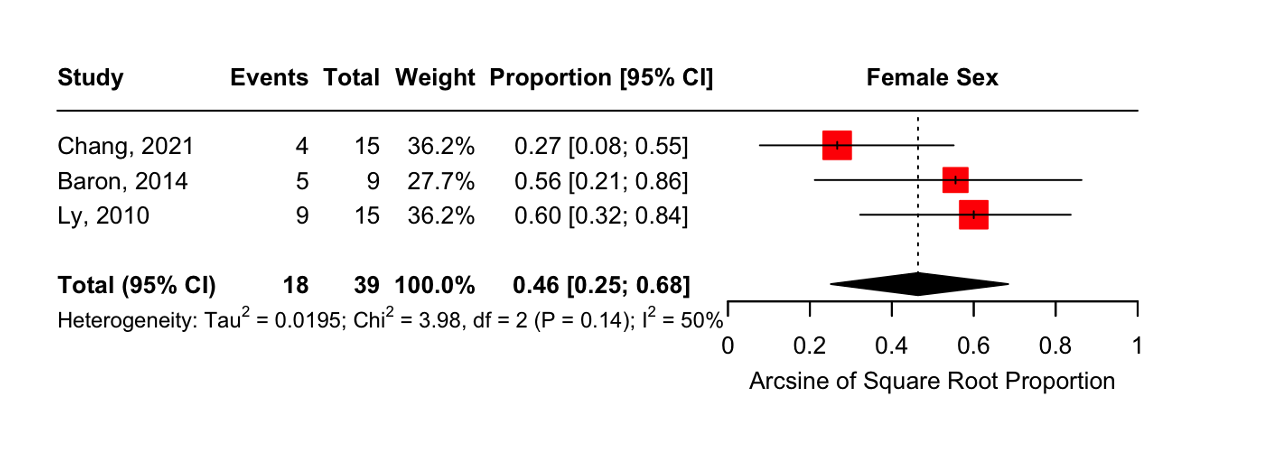
**

**Figure S17.:** Ratios for frontal-to-global amyloid PET distribution among patients with CAA and AD (Panel a), and among patients with CAA and HC (Panel b).


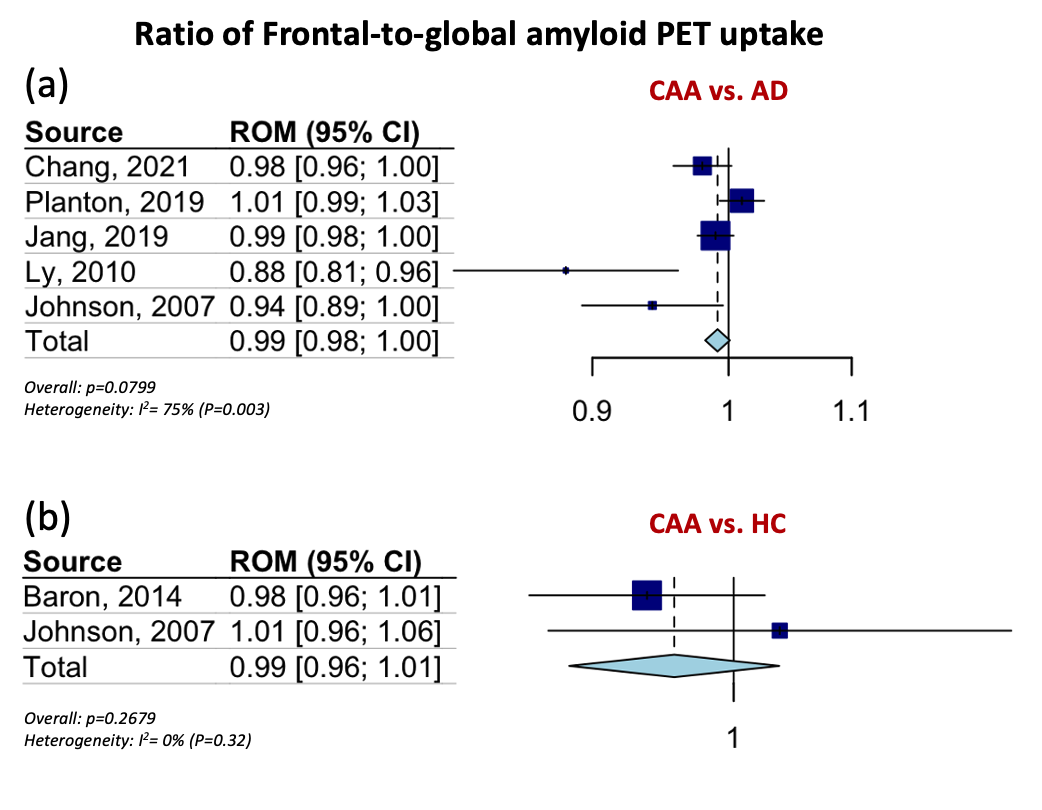


**Figure S18.:** Ratio for frontal amyloid PET distribution among patients with CAA and hypertensive ICH.


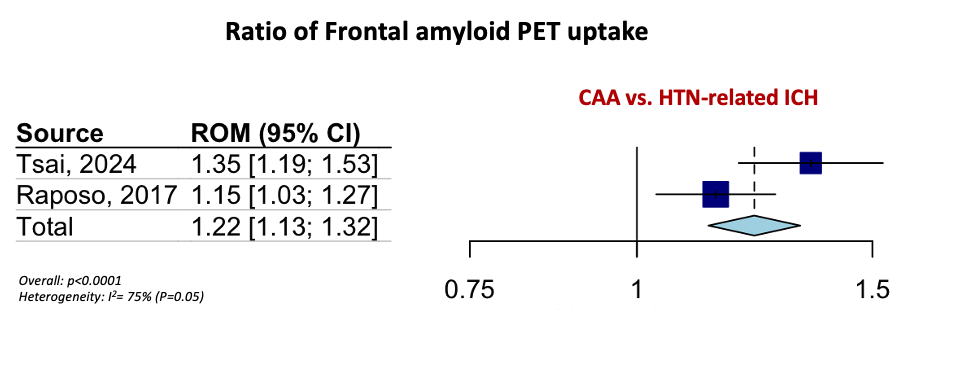


**Figure S19.:** Ratio for global amyloid PET distribution among patients with CAA and mixed pathology.


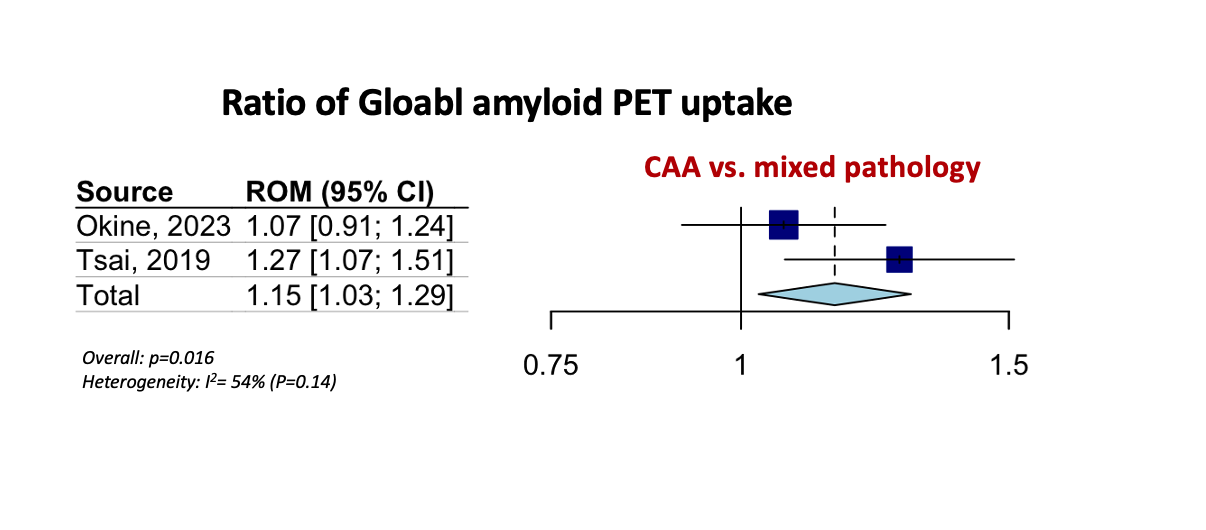


**Figure S20.:** Ratio for frontal amyloid PET distribution among patients with hypertensive ICH and mixed pathology.


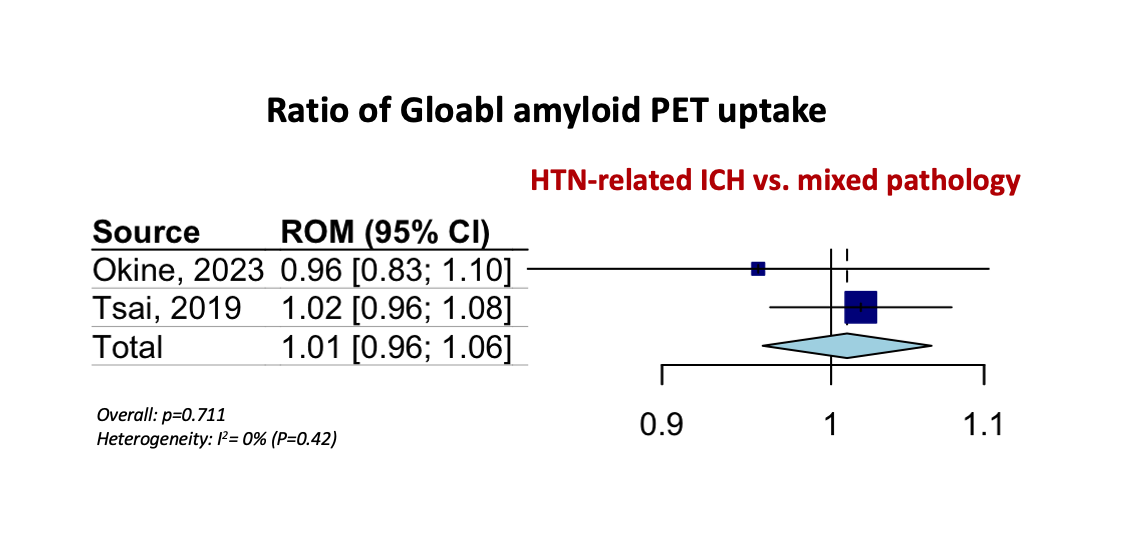


**References used in the Supplement.**

1. Kuo PY, Tsai HH, Lee BC, et al. Differences in lobar microbleed topography in cerebral amyloid angiopathy and hypertensive arteriopathy. Sci Rep. 2024;14(1):3774.
2. Tsai HH, Liu CJ, Lee BC, et al. Cerebral tau pathology in cerebral amyloid angiopathy. Brain Commun. 2024;6(2):fcae086.
3. Okine DN, Knopman DS, Mosley TH, et al. Cerebral Microbleed Patterns and Cortical Amyloid-β: The ARIC-PET Study. Stroke. 2023;54(10):2613-2620.
4. Jo S, Cheong EN, Kim N, et al. Role of White Matter Abnormalities in the Relationship Between Microbleed Burden and Cognitive Impairment in Cerebral Amyloid Angiopathy. J Alzheimers Dis. 2022;86(2):667-678.
5. Chang Y, Liu J, Wang L, et al. Diagnostic Utility of Integrated11C-Pittsburgh Compound B Positron Emission Tomography/Magnetic Resonance for Cerebral Amyloid Angiopathy: A Pilot Study. Front Aging Neurosci. 2021;13:721780.
6. Planton M, Pariente J, Nemmi F, et al. Interhemispheric distribution of amyloid and small vessel disease burden in cerebral amyloid angiopathy-related intracerebral hemorrhage. Eur J Neurol. 2020;27(8):1664-1671.
7. Planton M, Saint-Aubert L, Raposo N, et al. Florbetapir Regional Distribution in Cerebral Amyloid Angiopathy and Alzheimer's Disease: A PET Study. J Alzheimers Dis. 2020;73(4):1607-1614.
8. Jang H, Jang YK, Kim HJ, et al. Clinical significance of amyloid β positivity in patients with probable cerebral amyloid angiopathy markers. Eur J Nucl Med Mol Imaging. 2019;46(6):1287-1298.
9. Tsai HH, Pasi M, Tsai LK, et al. Microangiopathy underlying mixed-location intracerebral hemorrhages/microbleeds: A PiB-PET study. Neurology. 2019;92(8):e774-e781.
10. Raposo N, Planton M, Péran P, et al. Florbetapir imaging in cerebral amyloid angiopathy-related hemorrhages. Neurology. 2017;89(7):697-704.
11. Tsai HH, Tsai LK, Chen YF, et al. Correlation of Cerebral Microbleed Distribution to Amyloid Burden in Patients with Primary Intracerebral Hemorrhage. Sci Rep. 2017;7:44715.
12. Gurol ME, Becker JA, Fotiadis P, et al. Florbetapir-PET to diagnose cerebral amyloid angiopathy: A prospective study. Neurology. 2016;87(19):2043-2049.
13. Baron JC, Farid K, Dolan E, et al. Diagnostic utility of amyloid PET in cerebral amyloid angiopathy-related symptomatic intracerebral hemorrhage. J Cereb Blood Flow Metab. 2014;34(5):753-8.
14. Gurol ME, Viswanathan A, Gidicsin C, et al. Cerebral amyloid angiopathy burden associated with leukoaraiosis: a positron emission tomography/magnetic resonance imaging study. Ann Neurol. 2013;73(4):529-36.
15. Ly JV, Donnan GA, Villemagne VL, et al. 11C-PIB binding is increased in patients with cerebral amyloid angiopathy-related hemorrhage. Neurology. 2010;74(6):487-93.
16. Johnson KA, Gregas M, Becker JA, et al. Imaging of amyloid burden and distribution in cerebral amyloid angiopathy. Ann Neurol. 2007;62(3):229-34.
17. Guyatt GH, Oxman AD, Schünemann HJ, et al. GRADE guidelines: a new series of articles in the Journal of Clinical Epidemiology. J Clin Epidemiol 2011; 64: 380–382.
18. Meretoja A, Strbian D, Putaala J, et al. SMASH-U: a proposal for etiologic classification of intracerebral hemorrhage. Stroke. 2012;43(10):2592-7.
19. Sterne JA, Hernán MA, Reeves BC, et al. ROBINS-I: a tool for assessing risk of bias in non-randomised studies of interventions. Bmj 2016; 355: i4919.
